# Induction of antigen specific intrahepatic CD8+ T cell responses by a secreted heat shock protein based gp96-Ig-PfCA malaria vaccine

**DOI:** 10.3389/fimmu.2023.1130054

**Published:** 2023-03-28

**Authors:** Laura Padula, Eva Fisher, Wathsala Wijayalath, Noelle B. Patterson, Jun Huang, Harini Ganeshan, Tanisha Robinson, François A. Bates, Margaret A. Hanson, Monica L. Martin, Katelyn Rivas, Denisse Garcia, Kimberly A. Edgel, Martha Sedegah, Eileen Villasante, Natasa Strbo

**Affiliations:** ^1^Department of Microbiology and Immunology, University of Miami Miller School of Medicine, Miami, FL, United States; ^2^Malaria Department, Naval Medical Research Center (NMRC), Silver Spring, MD, United States; ^3^CAMRIS International, Bethesda, MD, United States; ^4^Henry M. Jackson Foundation for the Advancement of Military Medicine, Inc. (HJF), Bethesda, MD, United States; ^5^Malaria Serology Lab, Immunology Core, Walter Reed Army Institute of Research (WRAIR), Silver Spring, MD, United States; ^6^Parsons Technical Services Inc., Pasadena, CA, United States; ^7^Animal Medicine Branch, Veterinary Services Program, Walter Reed Army Institute of Research (WRAIR), Silver Spring, MD, United States; ^8^Necropsy Branch, Veterinary Services Program, Walter Reed Army Institute of Research (WRAIR), Silver Spring, MD, United States

**Keywords:** Gp96, malaria, vaccine, liver CD8+ T cells specific, memory

## Abstract

**Introduction:**

A highly efficacious and durable vaccine against malaria is an essential tool for global malaria eradication. One of the promising strategies to develop such a vaccine is to induce robust CD8+ T cell mediated immunity against malaria liver-stage parasites.

**Methods:**

Here we describe a novel malaria vaccine platform based on a secreted form of the heat shock protein, gp96-immunoglobulin, (gp96-Ig) to induce malaria antigen specific, memory CD8+ T cells. Gp96-Ig acts as an adjuvant to activate antigen presenting cells (APCs) and chaperone peptides/antigens to APCs for cross presentation to CD8+ T cells.

**Results:**

Our study shows that vaccination of mice and rhesus monkeys with HEK-293 cells transfected with gp96-Ig and two well-known *Plasmodium falciparum* CSP and AMA1 (PfCA) vaccine candidate antigens, induces liver-infiltrating, antigen specific, memory CD8+ T cell responses. The majority of the intrahepatic CSP and AMA1 specific CD8+ T cells expressed CD69 and CXCR3, the hallmark of tissue resident memory T cells (Trm). Also, we found intrahepatic, antigen-specific memory CD8+ T cells secreting IL-2, which is relevant for maintenance of effective memory responses in the liver.

**Discussion:**

Our novel gp96-Ig malaria vaccine strategy represents a unique approach to induce liver-homing, antigen-specific CD8+ T cells critical for *Plasmodium* liver-stage protection.

## Introduction

Malaria remains one of the deadliest parasitic diseases causing over half a million deaths worldwide every year (1) https://www.who.int/teams/global-malaria-programme/reports/world-malaria-report-2021). Development of a highly efficacious malaria vaccine is thus crucial. Many of the malaria vaccine strategies aim to prevent infection and clinical disease by disrupting malaria liver-stage parasite development. However, the RTS,S malaria vaccine, which was recently recommended by the WHO for use in preventing *Plasmodium falciparum* malaria in children living in regions with moderate to high transmission, provides only a short-lived, partial protection against malaria (2, 3). Lack of vaccine-induced, persistent, and robust T cell-mediated immune responses may be partly accountable for this failure (4, 5).

*Plasmodium* antigen-specific CD8+ T cells play a major role in protection against liver-stage malaria parasites (6–8). In fact, a non-circulating population of antigen-specific, memory CD8+ T cells that permanently reside in the liver is seemingly critical for liver-stage parasite clearance (9, 10). These liver resident memory CD8+ T cells share the phenotypic markers of tissue resident memory T (Trm) cells, associated with tissue retention (CD69), liver homing (CXCR3/CXCR6) and memory differentiation (low KLRG1). Liver Trm cells constantly patrol the liver sinusoids, potentially acting as sentinels against hepatic re-infection by malaria parasites (11). Liver localized immune surveillance mediated by Trm cells is now believed to be critical for a rapid recall response against infected hepatocytes. Therefore, several efforts have been made to trap malaria-specific CD8+ Trm cells permanently in the liver after initial priming in the secondary lymphoid organs (12–14). CD8+ T cell adoptive transfer experiments show induction of liver Trm cells following vaccination with radiation-attenuated sporozoites (RAS) (9). Anti-CXCR3-mediated depletion of liver Trm cells completely abrogates the protection induced by RAS and prime-trap malaria vaccination (9). Likewise, large number of antigen-specific, CD8+ Trm cells induced by prime-trap vaccination appears to correlate with malaria liver-stage protection. Therefore, searching for novel strategies facilitating liver recruitment and residency of antigen-specific memory CD8+ T cells may strengthen current malaria vaccine development efforts.

In the recent years, a secreted form of the heat-shock protein gp96, gp96-Ig, has been used as a novel and effective platform to induce robust tissue-specific, memory CD8+ T responses (15–19). Gp96 acts as a biological adjuvant that activates antigen presenting cells *via* toll-like receptor TLR2 and TLR4 (20, 21). At the same time, gp96 can specifically deliver (chaperone) antigens to dendritic cells (DCs) *via* CD91-receptor mediated endocytosis (22–26). These antigens are directed for MHC-class I cross-presentation, leading to co-stimulatory molecule mediated cross-priming and activation of antigen-specific, effector CD8+ CTLs (24, 25). In our previous studies, we demonstrated that vaccination with HEK-293 cells secreting gp96-Ig (293-gp96-Ig) that chaperones femto-molar concentrations of various antigens including ovalbumin (OVA), and the pathogen antigens HIV/SIV gag, Retanef (RTN), and envelope (env), and SARS-CoV-2 glycoprotein S have effectively induced antigen-specific, CD8+ T cells in tissues (18, 19). The 293-gp96^SIV^-Ig induced SIV-specific mucosal memory CD8+ T cell responses seem to play a critical role in protecting rhesus macaques against a highly pathogenic strain of SIV (18, 27–29). Here, we exploited multiple properties of the gp96-Ig molecule to see whether this approach can effectively induce *Plasmodium* antigen-specific, liver-resident, memory CD8+ T cells in mice and rhesus macaques. We selected two well-known *Plasmodium falciparum* (Pf) antigens, circumsporozoite protein (PfCSP) and apical membrane antigen 1 (PfAMA1), for this proof-of-concept study. Here, we show that the HEK-293 cell-based, gp96-Ig-PfCSP (C)-PfAMA (A) (293-gp96-Ig-PfCA) vaccine can elicit PfCSP- and PfAMA1-specific, effector memory and tissue resident memory CD8+ T cell responses in liver. New evidence about the significant contribution of liver-resident Pf antigen-specific CD8+ T cell responses in Pf pre-erythrocytic stages immunity (8), highlights the significance and need for further development of innovative vaccine approaches, such as secreted gp96-Ig technology.

## Materials and methods

### Generation of 293-gp96-Ig-PfCA vaccine cells

Human embryonic kidney (HEK)-293 cells, obtained from the American Tissue Culture Collection (ATCC), were transfected with 3 plasmids: B45 (expressing gp96-Ig, University of Miami, UM), pcDNA3.1 encoding PfAMA1 gene (originated from VR2571 plasmid encoding PfAMA1 gene, Naval Medical Research Center, NMRC) and pcDNA3.1 encoding PfCSP gene (originated from VR2577 plasmid encoding PfCSP gene, NMRC) (30). The B45 plasmid is a bovine papilloma virus derived vector from which the potentially transforming early genes E5, E6, E7, and late genes L1 and L2 have been removed. The VR1020 plasmid backbone (Vical, Inc., San Diego, CA) expresses malaria transgenes codon-optimized for expression in mammalian cells of either the PfCSP transgene (PfCSP 3D7 modified by deletion of 16 central repeat sequences, a TPA signal sequence added and a 23aa tail that increases expression) or the PfAMA1 transgene (PfAMA1 3D7 ectodomain with native signal sequence replaced with the TPA signal sequence), respectively under the human CMV-IE enhancer/promoter (30). All 293 cells were transfected using Effectene (QIAGEN, Valencia, CA) following the manufacturers’ protocols. Controls cells were transfected with B45 plasmid expressing secreted gp96-Ig (293-gp96-Ig mock control, no malaria antigens present). Transfected cells were cultured under selection medium containing G418 (1mg/ml) and Zeocin™ (50 µg/ml). After the stable transfection cell line was established, single cell cloning was performed and all the cell clones were first screened for gp96-Ig production and then for malaria antigen expression. Cells were irradiated with 120 Gy in a cobalt (Co) irradiator and stored frozen in cryopreservation media containing 25% human serum albumin and 10% DMSO until use as vaccine cells for immunization. Vaccine cells sterility testing by IMPACT II PCR was performed and all test results were negative.

### Gp96-Ig levels and western blotting

**Figure 1:**
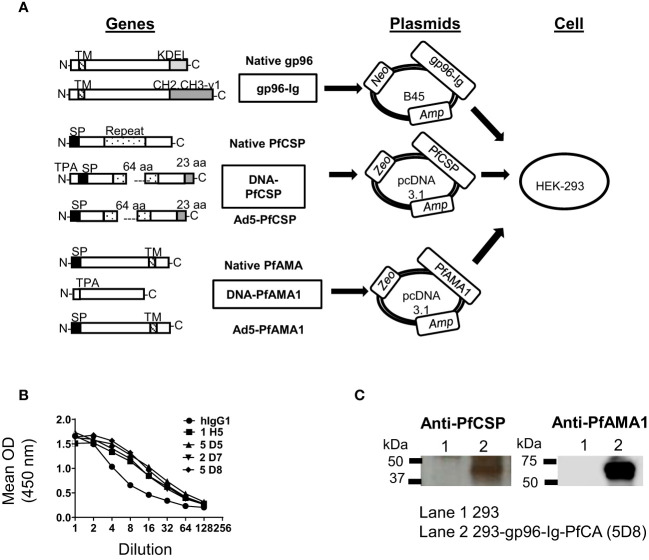
Generation of 293-gp96-Ig-PfCA vaccine cells. **(A)** Gene arrangement of native and modified proteins of gp96-Ig, PfCSP and PfAMA1 vaccine antigens. DNA-PfCSP and DNA-PfAMA1 represent the modified gene constructs used to generate 293-gp96-Ig-PfCA vaccine cells. Modified genes were inserted into the respective expression plasmids. Zeocin™ (Zeo), Neomycin (Neo) and Ampicillin (Amp) represent selectable markers. All 3 plasmids were transfected into HEK-293 to generate 293-gp96-Ig-PfCA vaccine cells. Both DNA and Ad5 constructs of PfCSP and PfAMA1 had been used to generate DNA/Ad5-PfCA vaccine, which has been described elsewhere (30, 31). DNA/Ad5-PfCA vaccine was used as the control in non-human primate experiments in the present study. KDEL – ER retention sequence of gp96; CH2, CH3-γ1 = hinge region and constant heavy chains (CH2 and CH3) of human IgG1; N = amino terminus; C = carboxy terminus; SP = Signal peptide; TPA = human tissue plasminogen activator signal sequence; Repeat = repeat region; aa = amino acids; TM = transmembrane domain. **(B)** Secretion of gp96-Ig by transfected HEK-293 cells (cell clones shown). One million cells were plated in 1 ml for 24h and gp96-Ig levels in the supernatant were determined by ELISA using anti-human IgG antibody. **(C)** Confirmation of protein expression by western blot. 293-gp96-Ig-PfCA cell lysates were analyzed by SDS-PAGE and Western blotting using anti-PfCSP and anti-PfAMA1 primary antibodies.

One million transfected HEK-293 cells were plated in 1 ml for 24h and gp96-Ig levels were determined in the supernatant by ELISA using 10 μg/ml goat anti-human IgG antibody (Jackson ImmunoResearch) for detection and human IgG1 Kappa (Jackson ImmunoResearch) as a standard (**Figure 1B**). Protein expression was verified by SDS-PAGE and Western blotting using rabbit anti-P. falciparum CSP protein antibody (CSP 207-397, Alpha Diagnostic International) at 1/1000 dilution and rabbit anti-P. falciparum AMA-1 protein (Cusabio) at 1/200 dilution. Binding of the primary antibodies was detected with HRP conjugated anti rabbit IgG (Jackson ImmunoResearch) at 1/5000 dilution. Protein bands were visualized by an enhanced chemiluminescence detection system (Amersham Biosciences, Piscataway, NJ) (**Figure 1C**).

### Animals and vaccination schedule

Mice used in this study were colony-bred mice (C57Bl/6) purchased from JAX Mice, The Jackson laboratory (Bar Harbor, ME). The animals were housed and handled in accordance with the standards of the Association for the Assessment and Accreditation of Laboratory Animal Care, International (AAALAC) under University of Miami Institutional Animal Care and Use Committee (IACUC) approved protocol. All mice (equal number of female and male mice in each experiment) were used at 6-10 weeks of age.

**Figure 2:**
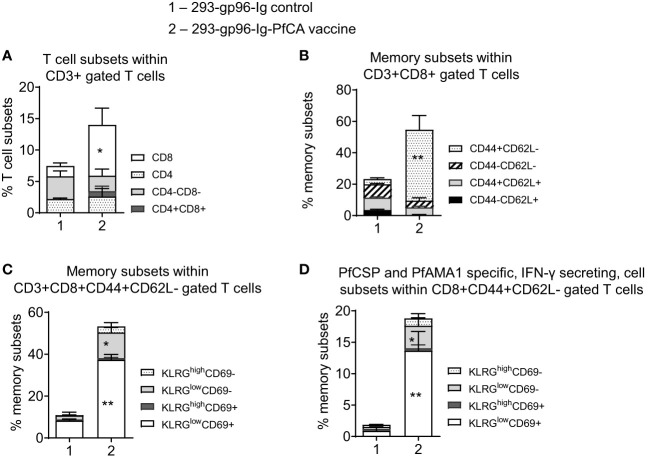
A single dose of 293-gp96-Ig-PfCA induces liver-infiltrating CD8+ T cell responses in mice. Characterization of intrahepatic memory CD8+ T cell responses in C57BL/6 mice, five days after a single dose of 293-gp96-Ig-PfCA vaccine or 293-gp96-Ig mock control *via* SC route. Intrahepatic lymphocytes stained with various T cell and memory surface markers were analyzed by flow cytometry to determine the **(A)** frequency of intrahepatic CD3+ T cell subsets. **(B)** frequency of memory CD3+CD8+ T cell subsets expressing CD44+/- and CD62L+/-. **(C)** frequency of CD3+CD8+CD44+CD62L- T cell subsets expressing KLRG1^low/high^ and CD69+/-. **(D)** To determine antigen-specific responses, total intrahepatic lymphocytes were cultured overnight in medium only or stimulated with one pool of overlapping PfCSP and one pool of overlapping PfAMA1 peptides. Following surface and intracellular staining, cells were analyzed by flow cytometer to determine the frequency of KLRG^low/high^ and CD69+/- cells within CD3+CD8+CD44+CD62L- cells secreting IFN-γ. Data represents mean ± standard deviation. Asterisks (*) denote significant differences between vaccinated mice (n=6) and mock controls (n=6) for a particular T cell subset at 0.05 alpha level. *p < 0.05, **p < 0.01.

Equivalent number of 293-gp96-Ig, 293-gp96-Ig-PfAMA1-PfCSP cells that produce 250ng/ml gp96-Ig or PBS were injected by subcutaneous (SC) intradermal (ID), intramuscular (IM) and intraperitoneal (IP) route in C56Bl/6 mice. For experiments in **Figure 2A** and **Supplementary Figure S1** mice were sacrificed 5 days after vaccination and for **Figures 2B–D** mice were vaccinated with 250 ng/ml 293-gp96-PfCA vaccine cells at week 0, 4 and 12. Mice were euthanized and spleen and liver were collected and processed into single-cell suspension.

Non-human primates used in this study were housed and handled in accordance with Animal protocol reviewed and approved by Walter Reed Army Institute of Research (WRAIR)/Naval Medical Research Center (NMRC) Institutional Animal Care and Use Committee in compliance with all applicable federal regulations governing the protection of animals and research. The experiments reported herein were carried out in compliance with the Animal Welfare Act and per the principles set forth in the “Guide for Care and Use of Laboratory Animals,” Institute of Laboratory Animals Resources, National Research Council, National Academy Press, 2011, the Public Health Service Animal Welfare Policy, and the policies of WRAIR.

Twenty adult rhesus macaques of Indian origin were housed at the WRAIR animal facility, two females ages 8 and 12 years old and one male age 8 years old were colony bred in Alice, Texas. The remaining 17 animals were from California National Primate Research Center, University of California, Davis, eight females and nine males both ranging 4-7 years old. All non-human primates were tested seronegative for Macacine herpesvirus 1, measles, simian retrovirus, simian immunodeficiency virus (SIV), simian T cell leukemia virus, and tuberculin skin test, and had pre-screen IFA titers of ≤ 1/80 (Pf3D7, blood stage; 3 animals negative or 1/10 titers, 14 animals 1/20 titer, 2 animals 1/40 titer, 1 animal 1/80 titer) and ≤ 1/40 (Pf3D7 sporozoite; 19 animals negative, 1 animal 1/40 titer). Animals were socially pair-housed with same sex and fed a commercial diet (Lab diet 5038, Purina Mills International), provided free access to water, and supplemented with a variety of fresh fruits and vegetables. Environmental enrichment was provided in accordance with standard operating procedures of the WRAIR animal facility. Animal cages were cleaned daily and sanitized bimonthly. Automatic lighting was provided through a 12:12 hour cycle.

A total of 20 Mamu-A*01+, malaria-naïve females and males rhesus macaques were enrolled in the study (**Supplementary Figure S3**) Group 1: received 293-gp96-IgPfCSP-PfAMA1, a cell based malaria vaccine expressing gp96, CSP and AMA1; 3 vaccinations at Week 0, 5 and 25; n=10. Vaccine cells, 40 x106 irradiated 293-gp96-Ig-PfCA cells that secrete 1 μg of gp96-Ig/million cells/24h were delivered by subcutaneous route (in two adjacent injection sites). Group 2: received NMRC-M3V-D/Ad-PfCA (30), a gene based DNA/Ad5 heterologous prime/boost vaccination: an equal mixture of two DNA plasmids encoding PfCSP and PfAMA1 (GMP lot, Aldevron, LLC) was delivered as prime, with three 2.0 mg intramuscular doses at week 0, 4, 8 followed by boosting with an Ad5 vector, 2 x 1010 pu (clinical seed stock, GenVec, Inc.) dose given once intramuscularly as an equal mixture of two non-replicating recombinant human serotype 5 adenovirus vectors expressing PfCSP and PfAMA1 antigens at 25 weeks; n=10.

**Figure 3:**
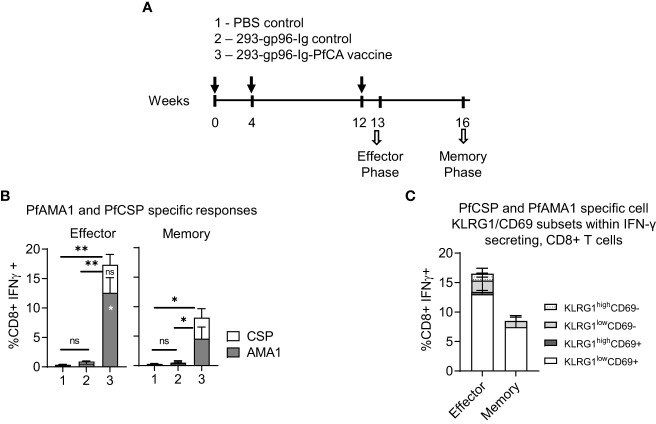
293-gp96-Ig-PfCA vaccinated mice induce antigen-specific, intrahepatic memory CD8+ T cells. **(A)** Characterization of antigen-specific, intrahepatic memory CD8+ T cell responses in C57BL/6 mice at effector (5 days post-last dose) or memory phase (4 weeks post-last dose). The mice received three doses 293-gp96-Ig-PfCA vaccine secreting 250ng/mL of gp96-Ig at week 0, 4 and 12 *via* SC route. Control mice received gp96-Ig alone, mock cells secreting 250ng/mL of gp96-Ig, or PBS. Total intrahepatic lymphocytes were cultured overnight in medium only or with a pool of overlapping PfCSP and PfAMA1 peptides. Following surface and intracellular staining, cells were analyzed by flow cytometry to determine the frequency of PfAMA1 and PfCSP specific **(B)** CD8+ T cells secreting IFN-γ. **(C)** KLRG1^low/high^ and CD69+/- cells within CD3+CD8+ T cells secreting IFN-γ. Data represents mean ± standard deviation. Asterisks (*) denote significant differences between vaccinated mice (n=6) and mock controls (n=6) for a particular T cell subset at 0.05 alpha level. Asterisks inside the column denote difference between peptide-(PfAMA1 and PfCSP) specific effector and memory responses.

### Isolation of lymphocytes from spleen, blood and liver tissue

For mouse samples, mice were euthanized in line with the 2013 AVMA panel on Euthanasia, spleens and livers were collected and tissues processed into single cell suspension. Splenocytes were treated with ACK lysis buffer (Thermo Fisher Scientific, Waltham, MA) to lyse red blood cells. Mouse livers were first divided into 4 equal pieces and processed in a separate 50 ml tube. Each liver piece was cut into small pieces and cells were gently forced through a 70 µM cell strainer using a 10 cc syringe plunger, with 25-30 mL of complete medium. Hepatocytes were separated from intrahepatic lymphocytes (IHL) by centrifugation at 60g (rcf) for 1 min. Supernatants containing IHL were collected and re-suspended in 36% Percoll PLUS solution (Cytiva life science, Marlborough, MA) and centrifuged at 2000 rpm for 30 min at room temperature. The upper layer containing hepatocytes was removed and the cell pellet containing IHL was collected. ACK lysis buffer was added to lyse the red blood cells. Isolated IHL were resuspended in complete medium and used for intracellular cytokine staining.

For non-human primate terminal sampling, (4 female, 4 male) rhesus macaques were anesthetized with 10 mg/ml ketamine and 0.55 mg/kg acepromazine combination IM and blood drawn (BD vacutainers: BD 367986 gold top serum separator tubes and BD 367878 dark green top heparinized tubes). Animals were intubated, anesthetized with isoflurane, surgical prepped, and just before harvest of organs euthanized with sodium pentobarbital (SomnaSol) overdose 100 mg/kg). All organs were removed within 21 minutes; each organ was placed on ice within 5 minutes of harvest, the majority in 1 minute or less. Livers were first rinsed with 100-300 ml cold (2.0-6.0°C) commercially prepared transport media (Custodiol^®^ HTK, Essential Pharmaceuticals, LLC, Durham, NC) and place into a sterile sample bag (710 mL Whirl-Pak, Weber Scientific, Hamilton, NJ) containing 50-100ml of cold (2.0-6.0°C) transport media and shipped overnight from NMRC to UM.

The liver was first cut in 4 pieces with scissors and then in smaller size (1- 2 mm) fragments and passed through a 250 µm mesh, using a plunger to assist with the passage and achieve a better mechanical extraction and using Hank’s Balanced Salt Solution (HBSS) buffer for rinsing. Filtered suspension was passed one more time through 70 µm strainers and centrifuged for 1 minute at 60g to pellet hepatocytes. The supernatants containing intrahepatic lymphocytes were resuspended in 15 ml of Iscove’s Modified Dulbecco’s Medium (IMDM) with 10% fetal bovine serum and layered on Ficoll-Hypaque. After 20 min centrifugation at 2000 rpm with brake off, the interface layer contained the mononuclear cells that were immediately used for flow cytometry and intracellular cytokine assay.

For non-human primate survival sampling, 20 (10 female, 10 male) rhesus macaques were sedated with 10 mg/ml ketamine and 0.55mg/kg acepromazine combination IM and whole blood (up to 10% total blood volume, 16-31.5 ml/draw) was collected from the femoral vein directly into heparinized collection tubes (BD 367878, dark green top) for T cell analyses [flow cytometry (shipped overnight in gel-pack control ambient temperature) and FluoroSpot] or into serum separation tubes (BD 367986, gold/yellow top) for antibody analyses. Animals were returned to cages after sample collection and observed through recovery. Blood samples were placed on a rocker, room temperature, until shipped or processed. Mononuclear cells (PBMCs) were isolated by density-gradient centrifugation using Ficoll-Hypaque Plus (GE Healthcare). Serum samples (75 µl) were aliquoted into polypropylene micro tubes and stored at -800C, and later used to determine Pf-specific antibody titers.

### Intracellular cytokine staining

Spleen (mice only), PBMCs and intrahepatic lymphocytes from immunized animals were analyzed for PfAMA1- and PfCSP-specific CD8+ T cell responses. 1-1.5x10/6 cells were incubated for 12 h with pools of 15-meric peptides (1.25 µg/ml of each peptide) overlapping by 11 amino acids covering the entire PfAMA1 and PfCSP proteins, anti-CD28, anti-CD49d (BD Pharmingen, San Diego, CA) followed by addition of Brefeldin A (GolgiPlug; BD Bioscience) (10 µg/ml) for additional 6 h. Costimulation without peptides served as background control. The results are calculated as the total number of cytokine-positive cells with background (costimulation medium) subtracted. Specific responses were detected by intracellular cytokine staining as described in the Flow cytometry.

### Flow cytometry

Peptide stimulated intrahepatic lymphocytes isolated from mice were first labeled with live/dead detection kit (Yellow Amine or Violet Thermo Fisher Scientific) and then were stained for surface markers, fixed, permeabilized and stained for intracellular cytokines and molecules (28, 30) using combinations of the following fluorochrome-conjugated antibodies (all from BioLegend, San Diego, CA),: CD45 30-F11 (CD45; APC-Cy7), CD3 17A2 (CD3; AF700), CD4 RM4-5 (CD4; PE Cy7), CD8 53-6.7 (CD8; PerCP and Spark Blue 550), CD69 H1.2F3 (CD69; PE Dazzle 594), CD44 IM7 (CD44; BV711 and BV605), CD62L MEL-14 (CD62L; BV 605 and BV510), KLRG1 2F1 (KLRG1;PE), IFNg XMG1.2 (IFNg; AF 488).

For non-human primate PBMCs and intrahepatic lymphocytes: CD3 SP34-2 (CD3; Alexa700), CD4 L200 (CD4; BV605), CD8 SK-1 (CD8; BV650), CD69 FN50 (CD69; PE-Dazzle 594), CXCR3 G025H7 (CXCR3; BV711), CD103 Ber-ACT8 (CD103; PE-Cy7 and AF647), Granzyme B GB11 (Granzyme B; Pac Blue and PerCP-Cy5.5), Perforin dG9 (Perforin: BV510), TNFα MAB11 (TNFα; FITC), IFNγ B27 (IFNγ; APC), IL-2 JES6- 5H4 (IL-2; PE), Live/Dead kit (Thermo Fisher). Antibodies were purchased from BD Pharmingen, BD Bioscience, and BioLegend. Data was collected on an Fortessa (BD Biosciences). Analysis was performed using FlowJo 10 software (Tree Star). First cells were gated on live cells and then lymphocytes were gated for CD3+ and progressive gating on CD8+ T cell subsets. Antigen-responding CD8+T cells (IFN-γ or IL-2 or TNF-α producing/expressing cells) were determined either on the total CD8+ T cell population or on CD8+CD69+ cells.

Acquisition was limited to cells expressing Alexa700 fluorochrome/CD3 at a particle cut-off size (FSC) of 3000 and 50,000 events/sample were acquired at a medium flow rate by 20-color, Fortessa flow cytometer at the Sylvester Comprehensive Cancer Center Flow Core, UM facility using the FACS DIVA software. Flow data were analyzed by FlowJo 10 software.

### FluoroSpot assay

Antigen-specific circulating peripheral blood mononuclear cells (PBMC) secreting single or multiple cytokines were evaluated using pre-coated FluoroSpot plates and kits purchased from Mabtech (Mabtech AB, Nacka Strand, Sweden). Plates and kits were used according to manufacturer’s instructions. Freshly collected heparinized monkey blood was processed for PBMCs separation using Ficoll method. 4x105 PBMCs were suspended in 100 µL complete medium (RPMI-1640 supplemented with 1% Penicillin Streptomycin, 1% L-Glutamine [from GIBCO/Life Technologies Corporation, Grand Island, NY] and 10% Fetal Bovine Serum [SIGMA]) and incubated in FluoroSpot plates with stimulants comprised of synthetic peptides purchased from Mimotopes, Clayton, Victoria, Australia. Full-length PfCSP and PfAMA1 amino acid sequences were covered by a series of 15 amino acid (aa) peptides overlapping by 11 aa. The total number of 15 amino acids peptides pooled into a mega pool for PfCSP was 65 and PfAMA1 was 153 at a final concentration of 1.25 µg/mL. phytohemagglutinin (PHA), a mitogen from SIGMA was used as a positive control for cell viability. Negative control unstimulated PBMCs wells received medium only. Cultures were incubated for 40-42 h at 37°C in 5% CO2. Each PBMC sample was assayed in duplicates due to limitation of cells and the number of single IFNγ and IL-2-secreting cells were recognized as spot-forming cells (sfc) and enumerated using an automated FluoroSpot reader (AID iSpot, GmbH, Germany).

### Immunohistochemistry

Rhesus macaque liver tissue samples were snap frozen in O.C.T. compound (Tissue-Tek^®^, Sakura Finetek, Cat#: 4583) 24h after euthanasia and kept at -80°C wrapped in foil paper. Slides were prepared by making 8 micrometer cuts with a microtome and preserved at -80°C until ready to use. Slides were thawed at room temperature for 5 min and fixed in pure cold acetone (VWR chemicals, BDH^®^, Catalog#: BDH1101) for 10 min, followed by 3 washes (1xPBS/5min each). Tissue areas were demarked with PAP pen and slides were blocked with 1xPBS/5% Bovine Serum Albumin (BSA) at room temperature for 2h. The following fluorescent antibodies (Biolegend, San Diego, CA): anti-human 488-CD8a (Alexa Fluor, clone RPA-T8), anti-human CD69 (Alexa Fluor 647, clone FN50) and mouse IgG1, kappa, Isotype control (Alexa Fluor 647, clone P3) were added at 1:50 and 1:100 dilutions in 1xPBS/5% BSA and/or Isotype control diluted at 1:50, and incubated overnight at 4°C in dark under humid conditions. Next day, slides were washed 3 times for 5 min with 1xPBS, mounted with Prolong Gold anti-fade reagent with DAPI (Cat#36935, Invitrogen, Carlsbad, CA) and acquired on Keyance microscope (BZ-X Viewer) using the following filters; DAPI (for nuclear stain), GFP (AF488), Cy5 (AF647).

### Immunofluorescence assay

Teflon printed 12-well slides (Electron Microscopy Sciences, Hatfield, PA) were coated either with P. falciparum (3D7) sporozoites (5,000 sporozoites per well/2% bovine serum albumin (BSA, Sigma) or parasite infected-red blood cells (iRBC) prepared 1:20 dilution from the final suspension and dispensed 10 µl per well. Slides were air dried and stored at −80°C until ready to use. Upon thawing, slides were blocked with PBS/1% BSA for 30 min at 37°C. Serum at two-fold dilutions was added to the wells and incubated for 1 h at 37°C. Slides were washed three times with 1xPBS and incubated with fluorescein-labeled anti-monkey IgG (Seracare Life Sciences Inc, Milford, MA) secondary antibody for 30 min at 37°C. Washed slides were mounted with Vectashield-DAPI (Vector Laboratories, Burlingame, CA) and examined under Optical UV microscope. IFA results were reported as end point dilution representing last serum dilution at which fluorescence was scored as positive.

### Enzyme-linked immunosorbent assay

To assess vaccine induced PfCSP and PfAMA1 antibody responses, ELISAs were performed against recombinant full-length PfCSP (32) and PfAMA1 (3D7) (33) as the plate antigens. ELISAs were conducted as previously described (34–36). Briefly, serum samples collected from rhesus macaques at pre- and different time points post-vaccination were serially diluted, incubated with plate antigens and the bound primary antibodies were detected using Goat anti-human IgG (1:4000 dilution) and ABTS Peroxidase Substrate (KPL/Sera Care, Milford, MA). ELISA titers were defined as the serum dilution that resulted in OD=1 (414 nm).

### Statistical analysis

All experiments were conducted independently at least three times on different days. Appropriate statistical tests were applied for each comparison after determining the normality of the data by Shapiro-Wilk test and Kolmogorov-Smirnov tests at 0.05 alpha level. Comparisons of flow cytometry cell frequencies for mouse studies were measured by the two-way ANOVA test with Holm-Sidak multiple-comparison test, * p<0.05, ** p<0.01 and *** p<0.001 (**Supplementary Figure S1**) or using unpaired T-test (two-tailed) (**Figure 2**, **3**) ELISA titers were compared using unpaired T-test (two-tailed) (**Supplementary Figure S7D**) or Mann-Whitney U test (**Supplementary Figures S7C, B**). Welch’s correction was applied with unpaired T test, when P value of the F test to compare variances were ≤0.05. Data were presented as mean ± standard deviation in the text and in the figures. All statistical analysis were conducted at alpha 0.05 level using GraphPad Prism versions 8.0 for Windows (GraphPad Software, San Diego, CA, www.graphpad.com)

## Results

### HEK-293 vaccine cells express malaria antigens and secret gp96-Ig

Our vaccine was composed of live, irradiated, allogeneic/xenogeneic immortalized human embryonic kidney cells (HEK-293) stably transfected with three plasmids each encoding gp96-Ig, PfAMA1, or PfCSP proteins (hereinafter referred to as gp96-Ig-PfCA). The secretory form of gp96 protein (gp96-Ig), has been previously generated by replacing c-terminal, KDEL retention sequence of the human gp96 gene, with hinge region and constant heavy chains (CH2 and CH3) of mouse or human IgG1 (19, 37). The gp96-Ig gene was inserted into the B45 plasmid. B45 replicates as a multi-copy episome, encoding Ampicillin (Amp) and Neomycin (Neo) resistance protein and provides high levels of expression (37) (**Figure 1A**). The U.S. Food and Drug Administration (FDA) and the Office of Biotechnology Activities (OBA) have approved B45 plasmid encoding gp96-Ig fusion protein for human use (38, 39). Safety and immunogenicity of gp96-Ig based vaccines for the treatment of non-small cell lung cancer are currently being tested in clinical studies (clinical trial Identifier - NCT02117024, NCT02439450). The PfCSP gene has been modified by deleting 64 amino acids of the central repeat sequence and by adding a 23 amino acid segment from the transcriptional terminator of bovine growth hormone at the C terminus. Human tissue plasminogen activator (TPA) signal sequence has been added to the native signal sequence of PfCSP (**Figure 1A**) (30). The ectodomain of the PfAMA1 has been modified by replacing the native signal sequence with a TPA signal sequence (**Figure 1A****)**. Modified PfCSP and PfAMA1 genes were inserted into the mammalian plasmid pc DNA 3.1, encoding Ampicillin and Zeocin™ (Zeo) resistance proteins (**Figure 1A****)**. PfCSP and PfAMA1 genes were originally expressed in VR1020 plasmid (31, 40) as VR2571 and VR2577, respectively, GMP manufactured, authorized for evaluation under a U.S. FDA Investigational New Drug application (IND) and shown to be safe and immunogenic in the clinic (30).

293-gp96-Ig-PfCA vaccine cells were generated by stable co-transfection of HEK-293 cells with recombinant plasmids encoding gp96-Ig (B45), PfCSP and PfAMA1 (pcDNA3.1) antigens, one antigen per plasmid, in a stepwise approach (**Figure 1A**, **Material and Methods**). After establishing stable transfected cell lines resistant to both Neomycin and Zeocin™, we performed single cell cloning to select the cell clones with the highest production of secreted gp96-Ig. We confirmed by ELISA that clone 5D8 secretes gp96-Ig into culture supernatants at a rate of 2000 ng/mL/24h/10^6^ vaccine cells (**Figure 1B**). Our previous data indicate that gp96-Ig accumulation in cell culture supernatant is linear and time dependent (41, 42). We also have shown that irradiated gp96-Ig transfected cells were unable to replicate *in vitro*, but efficiently secrete gp96-Ig into culture supernatant similar to non-irradiated cells (17, 42). Presence of the transmembrane (TM) domain did not affect the secretion of gp96-Ig.

After confirming that clone 5D8 expresses PfCSP and PfAMA1, we used this clone in all immunogenicity studies as vaccine cells (**Figure 1C**). Expression of recombinant PfCSP (43kD) and PfAMA1 ectodomain (60 kD) by the vaccine cells was confirmed by analyzing vaccine cell lysates on sodium dodecyl-sulfate polyacrylamide gel electrophoresis (SDS-PAGE) and blotting with anti-PfCSP and PfAMA1 antibodies (**Figure 1C**).

The vaccination strategy is based on the quantity of gp96-Ig secreted by the vaccine cells to stimulate CD8+ CTL responses *via* APC-cross-presentation. The vaccination dose is therefore, standardized by the amount of gp96-Ig secreted by 1 x 10^6^ vaccine cells within 24 hours, *in vitro*. It was well established in our previous vaccine immunogenicity studies that the optimal dose for induction of antigen specific CD8+ T cell responses in mice is 250-500 ng/ml (17) and 10-20 µg/ml for non-human primates (18, 19). We found that the peak of effector responses after SC and IP vaccination is at day 5 (16, 17, 19, 37, 42).

### 293-gp96-Ig-PfCA induces, liver-infiltrating, antigen-specific memory CD8+ T cells in mice

We first determined the most effective route of vaccination, generating an optimal antigen-specific, tissue-infiltrating, CD8+ T cell response by 293-gp96-Ig-PfCA vaccine. A single dose of the vaccine (250 ng/ml) was delivered to mice *via* four different routes (**Supplementary Figure S1**). Mice were euthanized 5 days later and splenocytes or intrahepatic lymphocytes were stimulated with PfCSP or PfAMA1 peptide pools. Since interferon gamma (IFN-γ) is a key mediator of cytotoxic function of CD8+ T cells, we determined the frequency of antigen-specific, IFN-γ secreting CD8+ T cells to evaluate vaccine immunogenicity. We found significantly higher frequencies of PfCSP and PfAMA1 specific, IFN-γ secreting CD8+ T cells after vaccinating with intraperitoneal (IP) and subcutaneous (SC) routes compared to that of the intradermal (ID) and intramuscular (IM) routes (**Supplementary Figure S1****, p<0.01**). Mice immunized with 293-gp96-Ig alone or Phosphate Buffered Saline (PBS) showed only background level of antigen specific CD8+T cells (**Supplementary Figure S1**). These data showed that both IP and SC delivery of 293-gp96-Ig-PfCA vaccine cells can effectively induce liver-infiltrating, malaria antigen-specific CD8+ T cells. Since the translation of IP route of vaccination to the clinical settings could present a big hurdle, we decided to pursue subcutaneous (SC) delivery of 293-gp96-Ig vaccine in all immunogenicity studies described herein.

One major goal of an effective liver-stage malaria vaccine is to generate a repository of long-lasting, antigen-specific, memory CD8+ T cells in the liver, which will be readily available to fight potential malaria liver-stage infections (8). Therefore, we first characterized the phenotype of intrahepatic lymphocytes in mice, 5 days after a single dose of 293-gp96-Ig-PfCA vaccination (**Figures 2A–D**, **S2**). We found a significantly higher frequency of intrahepatic CD3+CD8+ T cells in vaccinated mice (p<0.05) compared to the mock controls (**Figures 2A**, **S2**). This intrahepatic, CD8+ T cell population in vaccinated mice were dominated by CD44+CD62L- effector phenotype, which was almost 9-fold higher than the CD44+CD62L+ central memory population (**Figures 2B**, **S2**).

We then analyzed CD44+CD62L- effector T cell population for expression of killer cell lectin-like receptor subfamily G member 1 (KLRG1) and CD69 markers to distinguish T cell memory phenotypes. Effector T cells expressing a high level of KLRG1 (KLRG1^high^) represent a population of terminally differentiated, short-lived effector T cells. Conversely, lower expression of KLRG1 (KLRG1^low^) on effector T cells mark them as proliferation-competent, long-lived memory-precursor effector cells. KLRG1^low^ effector T cells display an increased survival during the contraction phase of the immune response and differentiate into effector memory T cells (Tem) and tissue resident memory T (Trm) cells (43). High expression of the early T cell activation marker, CD69, signals for tissue retention of T cells. Therefore, CD69 serves as a canonical marker for tissue resident T cell phenotype in mice and humans (44, 45). CD69 is highly expressed on Trm cells in different tissues (46, 47) including the liver (9, 14).

After a single dose of 293-gp96-Ig-PfCA vaccine, a majority of CD44+CD62L- effector T cells in the liver had KLRG1^low^CD69+ phenotype (**Figure 2C**) indicating their commitment to become liver Trm cells. In addition, about 12% of the CD44+CD62L- effector T cells showed KLRG^low^CD69- phenotype (12.2 ± 2.3), representing a population of potential effector memory CD8+ T cells (**Figure 2C**). To evaluate antigen-specific, memory CD8+ T cell responses, we then stimulated intrahepatic lymphocytes with PfCSP and PfAMA1 peptide pools (PfCA) *in vitro*. Our data show that 18.8 ± 6.5% of the total CD8+CD44+CD62L- effector T cells secreted IFN-γ upon reactivation (**Figure 2D**). Also, vaccinated mice predominately exhibited KLRG1^low^CD69+ phenotype among PfCA-specific, CD44+CD62L- effector CD8+ T cells secreting IFN-γ (**Figure 2D**). In addition, a smaller population of PfCA-specific CD8+ IFN-γ+ T cells belonged to the KLRG^low^CD69- effector memory lineage (**Figure 2D**). Since CD69+ is also an early T cell activation marker, cells expressing CD69+ after *in vitro* stimulation, may contain a population of activated T cells as well. We however observed a KLRG^low^CD69+ Trm phenotype predominating in the livers of the vaccinated mice after direct phenotyping (without additional *in vitro* stimulation) (**Figure 2C**), indicating that most of our antigen specific CD69+ cells belong to the Trm phenotype and it is not result of CD69 expression after *in vitro* stimulation.

We next vaccinated the mice with three doses of 293-gp96-Ig-PfCA subcutaneously at 0, 4 and 12 weeks (**Figure 3A**). The frequency of PfCSP or PfAMA1- specific, IFN-γ secreting, intrahepatic CD8+T cells was evaluated at 5 days (effector phase) and 4 weeks (memory phase) after the last vaccination (**Figure 3A**). The effector phase had 17.3 ± 4.3% of CD8+ T cells secreting IFN-γ, specific to both antigens, which were significantly reduced at 4 weeks post-last vaccination (8.1 ± 3.4) (**Figure 3B**). However, CD8+IFN-γ+ responses were remarkably different between the two antigens, where a significantly higher proportion of the CD8+ T cells were specific to PfAMA1 in the effector phase (p<0.05). Interestingly, while the frequency of PfCSP specific CD8+IFNγ+ T cells remained unchanged, the frequency of PfAMA1, specific CD8+IFN-γ+ T cells showed a significant decrease in frequency (p<0.05) by the memory phase (**Figure 3B**). Our Western blot data show that transfected HEK-293 cells secrete a higher quantity of PfAMA1 than PfCSP protein *in vitro* (**Figure 1C**). Therefore, the quantity of each antigen secreted by HEK-293 cells may have affected the magnitude of the immediate effector responses, especially when one considers that the encoded PfAMA1 protein is larger (550 amino acids, aa) compared to the encoded PfCSP (389 aa).

KLRG1^low^CD69+ cells were predominant among the PfCA-specific, CD8+ IFN-γ+ T cells during effector and memory phase (**Figure 3C**). In the effector phase combined KLRG1^high^ CD69+ and CD69- cells and KLRG1^low^ CD69- cells make up to 20.7% of all PfCA specific CD8+IFN-γ + T cells (**Figure 3C**). However, in the memory phase these three cell subsets (KLRG1^high^ CD69+ and CD69- and KLRG1^low^CD69- effector memory cells) were present only in trace frequencies compared to effector phase (**Figure 3C**) (1.1% vs 0.3%), with overall contraction of 73% from the effector phase. The program of contraction in KLRG1^low^CD69+ cells, the predominant subset in the memory phase, was of much slower rate (47% loss of KLRG1^low^CD69+ cells from effector to memory phase) (**Figure 3C**). In addition, we did not see any substantial changes in the frequency of total KLRG1^low/high^CD69+/- subset, 5 days after three doses (16.5 ± 5.3) compared to a single dose of the 293-gp96-Ig-PfCA vaccine (18.8 ± 6.5) (**Figures 2D**, **3C**-effector phase).

Our data show that the 293-gp96-Ig-PfCA malaria vaccine induces a predominant population of liver-infiltrating CD44+CD62L-CD69+KLRG1^low^ Trm cells in mice, characterized by an increased production of IFN-γ upon *in vitro* antigenic stimulation. 293-gp96-Ig-PfCA vaccine induces a rapid liver infiltration of antigen-specific CD8+ T cells expressing CD44+CD62L-CD69+KLRG1^low^ Trm phenotype that persist during the memory phase. In addition, the long-term survival of the 293-gp96-Ig-PfCA induced antigen-specific CD8+IFN-γ+ T cells appears to be dependent on the particular malaria antigen.

### 293-gp96-Ig-PfCA induces liver-infiltrating, antigen-specific memory CD8+ T cells in rhesus macaques

**Figure 4:**
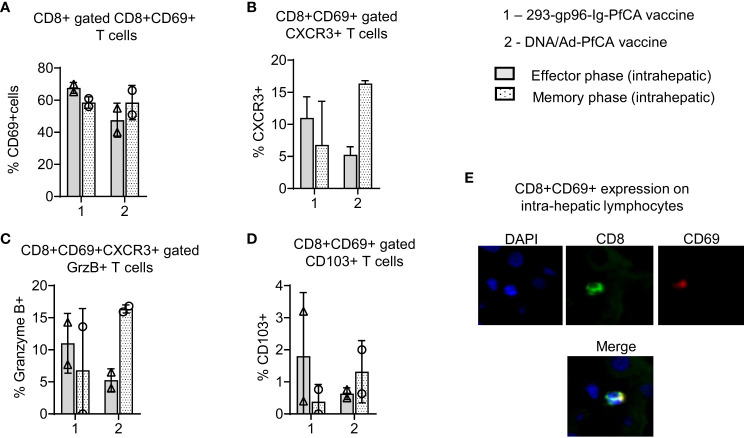
293-gp96-Ig-PfCA vaccinated rhesus macaques induce intrahepatic memory CD8+ T cells expressing Trm phenotype. Characterization of intrahepatic memory CD8+ T cell responses in rhesus macaques at effector (1 week, n=2) and memory (11 weeks, n=2) time-points following the third dose of the 293-gp96-Ig-PfCA vaccine. Control animals received DNA/Ad5-PfCA vaccine and were sacrificed at effector (3 weeks, n=2) and memory (11 weeks, n=2) time-points following the Ad5 boost. Optimal effector time-points of the two vaccines were determined based on the prior study results. Intrahepatic lymphocytes stained with the surface markers: CD3, CD8, CD69, CXCR3, CD103 and intracellular granzyme B (GrzB) were analyzed by flow cytometry to determine the frequency of **(A)** CD8+ gated CD8+CD69+ T cells **(B)** CD8+CD69+ gated CXCR3+ T cells **(C)** CD8+CD69+CXCR3+ gated GrzB+ T cells **(D)** CD8+ gated CD69+CD103+ T cells. **(E)** Frozen liver tissue sections (at week 26) of 293-gp96-Ig-PfCA vaccinated macaques showing expression of CD8 (green) and CD69 (red) with co-localization (yellow). Original magnification 40X with DAPI nuclear staining shown in blue. Data represent individual values and mean ± standard deviation.

**Figure 5:**
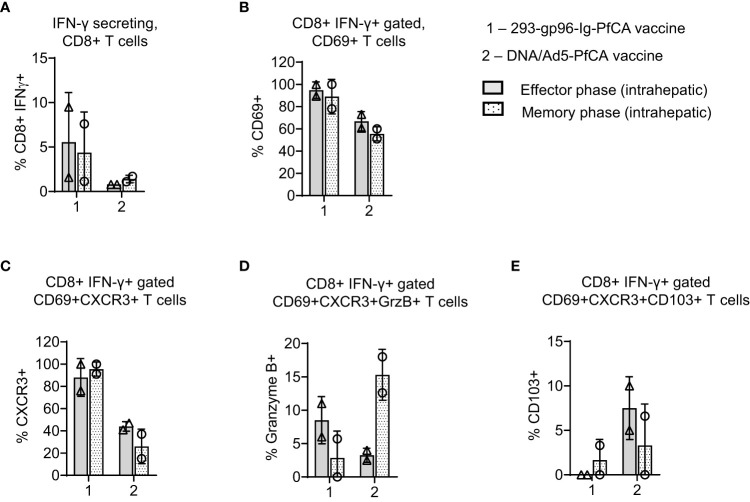
293-gp96-Ig-PfCA vaccinated rhesus macaques induce PfAMA1 specific CD8+ T cell memory responses in the liver. Characterization of PfAMA1-specific, intrahepatic memory CD8+ T cell responses in rhesus macaques at effector (1 week, n=2) and memory (11 weeks, n=2) time-points following the third dose of the 293-gp96-Ig-PfCA vaccine. Control animals received DNA/Ad5-PfCA vaccine and sacrificed at effector (3 weeks, n=2) and memory (11 weeks, n=2) time-points following the Ad5 boost. Optimal effector time-points of the two vaccines were determined based on the results of the prior studies. Intrahepatic lymphocytes were cultured overnight in medium only or with pool of overlapping PfAMA1 peptides. Cells were stained for surface CD3, CD8, CD69, CXCR3, CD103 and intracellular cytokine IFN-γ and granzyme **(B)** Cells were analyzed by flow cytometry to determine the frequency of **(A)** IFN-γ secreting CD8+ T cells **(B)** CD8+IFN-γ+ gated CD69+ **(C)** CD8+IFN-γ+ gated CD69+CXCR3+ **(D)** CD8+IFN-γ+ gated CD69+CXCR3+GranzymeB+ and **(E)** CD8+IFN-γ+ gated CD69+CXCR3+CD103+ cells. Data represent individual values and mean ± standard deviation.

We next evaluated the 293-gp96-Ig-PfCA vaccine induced cellular immune responses in the rhesus macaques (**Figures 4**; **S3**). The vaccine was administered *via* SC route, as it has shown the optimal responses in our mouse studies. The 293-gp96-Ig-PfCA vaccinated animals did not have any side-effects or adverse reactions to the vaccine. The DNA/Ad5-PfCA vaccine (plasmid DNA vectored PfCSP and PfAMA1, 3 dose prime followed by a human adenovirus serotype 5 vectored PfCSP and PfAMA1, 1 dose boost), administered IM, was used as the positive control (30). Although many useful immunological insights into malaria infection have been made by studying peripheral blood, it has become increasingly clear that a large proportion of malaria antigen specific cellular responses are enriched in the liver. We evaluated immunological changes within both peripheral blood and liver after 293-gp96-Ig-PfCA and DNA/Ad5-PfCA vaccinations. We observed a higher frequency of antigen specific CD8+ T cells within peripheral blood mononuclear cells (PBMCs) of DNA/Ad5-PfCA vaccinated animals compared to 293-gp96-Ig-PfCA vaccinated animals from 1 week (effector phase) and 11 weeks (memory phase) after the last dose of vaccination/boost as measured by intracellular cytokine staining of PBMCs following *in vitro* overnight PfCSP and PfAMA1 peptide pool stimulation (**Supplementary Figure S4A**). We also found that the 293-gp96-Ig-PfCA vaccine group had very few PBMCs secreting IFN-γ or IL-2 in response to PfCSP or PfAMA1 peptide pool stimulation, compared to the DNA/Ad5-PfCA animals as determined by FluoroSpot (**Supplementary Figures S4B, C**), during both effector and the memory phase. However, in one out of two analyzed macaques, the frequency of antigen specific CD8+ T cells within the liver lymphocytes was higher after 293-gp96-Ig-PfCA compared to DNA/Ad5-PfCA vaccination (**Figure 5A**), in both effector and memory phase, confirming our previous mouse and non-human primate findings about predominant tissue induction of antigen specific CD8+ T cell responses by gp96-Ig vaccines (18, 19).

Based on the data from our mouse studies, we assumed that gp96-Ig-PfCA vaccine would have induced CD8+ T cells preferentially infiltrating the liver. We found more than 60% of intrahepatic CD8+ T cells showing CD8+CD69+ Trm phenotype in 293-gp96-Ig-PfCA vaccinated rhesus macaques at 1 week (effector) post-vaccination (**Figure 4A**-effector, **Supplementary Figure 5**). This Trm population persisted up to 11 weeks (memory), indicating their ability to sustain in the liver for longer periods (**Figure 4A**-memory, **Supplementary Figure 5**). Likewise, we observed similar effector and memory CD8+CD69+ Trm cell frequencies in the two DNA/Ad5-PfCA vaccinated animals. About 60% of the effector phase CD8+CD69+ Trm cells expressed CXCR3, the liver-homing, interferon-inducible chemokine receptor, following 293-gp96-Ig-PfCA vaccination (**Figure 4B**-effector). The control DNA/Ad5-PfCA vaccine showed similar effector responses. Expression of CXCR3 on a larger proportion of CD8+CD69+ T cells indicates that these cells may have been recruited to the liver from their local site of origin. Also, they more likely do belong to the true liver CD8+ Trm phenotype, as CD69+ expression promotes the tissue retention of these liver CD8+ T cells. We also detected CXCR3 expression on CD8+CD69+ Trm cells harvested at 11 weeks post-vaccination (**Figure 4B**). Yet, the frequencies were lower compared to their effector time-point (**Figure 4B**). We then analyzed granzyme B expression on CD8+ T cells to assess the cytolytic potential (**Figure 4C**). About 7 to14% of CD8+CD69+CXCR3+ cells expressed granzyme B during the effector phase after the 293-gp96-Ig-PfCA vaccination (**Figure 4C**). Data were highly variable between the two animals during the memory phase, where one animal did not show any granzyme B expression. These data indicate that at least some Trm cells induced by gp96-Ig vaccination may have very low cytotoxic capacity during the resting memory phase (**Figure 4C**). In contrast, in the DNA/Ad5-PfCA vaccinated group, both animals showed a consistently higher frequency of granzyme B expressing Trm cells during the memory phase compared to that of the effector phase (**Figure 4C**). As shown in **Figure 4D**, memory responses of the two 293-gp96-Ig-PfCA vaccinated rhesus macaques showed a lower frequency of CD8+CD69+ T cells expressing CD103, a mucosal integrin expressed by a subset of Trm cells (48), compared to the vaccinated controls (**Figure 4D**).

To study antigen-specific, CD8+ T cell responses in the two rhesus macaques, intrahepatic lymphocytes were harvested at effector/memory time points post-vaccination and subsequently stimulated with a pool of PfAMA1 overlapping peptides (**Figures 5A–E**; **Supplementary Figure 6**). Frequency of PfAMA1-specific, CD8+IFN-γ+ T cells varied between the two 293-gp96-Ig-PfCA vaccinated animals (9.5% and 1.5%), without any substantial changes over time (**Figure 5A**). Almost, all the CD8+IFN-γ+ T cells showed CD69+ expression, both at effector (89% and 100%) and memory (78% and 100%) time-points post-293-gp96-Ig-PfCA vaccination, which were higher than that of the DNA/Ad5-PfCA vaccinated controls (**Figure 5B**). Likewise, at the effector time point of the 293-gp96-Ig-PfCA vaccination, almost all the (76% and 100%) PfAMA1 activated CD8+IFN-γ+ T cells co-expressed CD69+ and CXCR3 (**Figure 5C**). The memory recall responses at 11 weeks contained similar frequencies (91% and 100%) of IFN-γ+ secreting CD8+CXCR3+ T cells (**Figure 5C**). The PfAMA1-specific, CD8+IFN-γ+CD69+CXCR3+ T cell frequency was substantially lower in the two DNA/Ad5-PfCA vaccinated macaques and did not change overtime (**Figure 5C**). Interestingly, small fraction of CD8+IFN-γ+ T cells (11% and 6%) co-expressed CD69+CXCR3+ and granzyme B at the gp96-Ig-PfCA effector phase. This was further decreased by the memory phase, where one animal completely lacked granzyme B+ cells (**Figure 5D**). Surprisingly, the memory phase of the DNA/Ad5-PfCA vaccinated group had increased frequency of granzyme B+ cells (18% and 12.6%), which was comprised of a high proportion of CD8+IFN-γ+ T cells co-expressing CXCR3 (**Figures 5C, D**). DNA/Ad5-PfCA vaccinated rhesus macaques also had 5-10% of antigen activated CD8+IFN-γ+ T cells co-expressing CD69, CXCR3 and CD103 at the effector phase, where some cells were lost by the memory time-point. The gp96-Ig approach particularly lacked antigen-specific CD103 expression, where only one of the animals had few cells expressing CD103 during the memory phase (**Figure 5E**).

Even though our liver analysis was limited to two rhesus macaques, the data agree with the results from mouse studies described herein. Our data indicate that 293-gp96-Ig-PfCA vaccination can induce a persistent population of CD8+CD69+CXCR3 liver Trm cells, which may potentially elicit CXCR3-IFN-γ dependent effector functions upon reactivation. However, in the 293-gp96-Ig-PfCA group, the re-activated CD8+IFN-γ+CXCR3+ T cells appear to have a partially diminished cytotoxic potential, similar to resting liver CD8+ Trm cells (**Figure 5D**). In contrast, a majority of CXCR3+ expressing CD8+CD69+ Trm cells induced by the DNA/Ad5-PfCA vaccine do not seem to sustain for longer periods and more likely have poor CXCR3 mediated recall responses.

### 293-gp96-Ig-PfCA vaccine induces intrahepatic memory CD8+ IL-2+T cells

**Figure 6:**
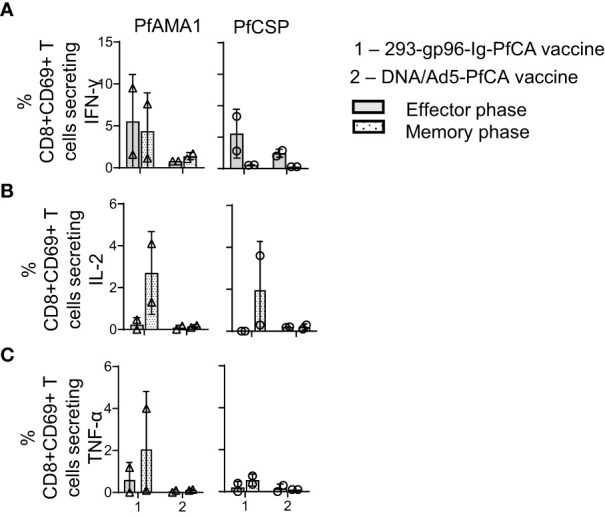
293-gp96-Ig-PfCA vaccine induces antigen-specific, intrahepatic memory CD8+ T cells secreting IL-2. Characterization of PfAMA1 and PfCSP-specific, cytokine responses by intrahepatic memory CD8+ T cell in rhesus macaques at effector (1 week, n=2) and memory (11 weeks, n=2) time-points following the third dose of the 293-gp96-Ig-PfCA vaccine. Control animals received DNA/Ad5-PfCA vaccine and sacrificed at effector (3 weeks, n=2) and memory (11 weeks, n=2) time-points following the Ad5 boost. Peripheral blood mononuclear cells (PBMCs) and intrahepatic lymphocytes were stimulated with PfCSP and PfAMA1 overlapping peptides for 18h and intracellular expression of **(A)** IFN-γ **(B)** IL-2 and **(C)** TNF-α in CD8+CD69+ cells were analyzed by flow cytometry. Data represent individual values and mean ± standard deviation.

We next compared PfAMA1 and PfCSP-specific IFN-γ, IL-2 and TNF-α responses after *in vitro* stimulation of intrahepatic lymphocytes (**Figures 6A–C**). In effector phase, one macaque had 9.5% of PfAMA1- and PfCSP-specific CD8+CD69+ T cells secreting IFN-γ after 293-gp96-Ig-PfCA vaccination, and the other macaque 1.59% (**Figure 6A**). In the memory phase, the frequency of PfAMA1-specific CD8+CD69+ T cells secreting IFN-γ was higher, in both macaques, than the frequency of PfCSP-specific CD8+CD69+ T cells. TNFα expression was very low for both Pf antigens in effector phase, while TNFα expression was the highest in memory phase in one 293-gp96-Ig-PfCA vaccinated animal (**Figure 6C**).

During the memory phase, the two 293-gp96-Ig-PfCA vaccinated animals had variable frequencies of IL-2 secreting CD8+CD69+T cells responding to AMA1 and CSP; however, both DNA/Ad5-PfCA vaccinated animals showed only background levels of IL-2-secreting CD8+CD69+ T cells during effector and memory phase (**Figure 6B**). In addition, the DNA/Ad5-PfCA vaccine failed to induce TNFα responses (**Figure 6C**).

In summary, contrary to blood (**Supplementary Figure S4A**), in the liver 293-gp96-Ig-PfCA vaccination induced higher frequency of IFN-γ expressing CD8+ T cells (**Figure 6A**) with co-expression of IL-2 for both Pf antigens in memory phase (**Figure 6B**).

### 293-gp96-Ig-PfCA vaccine induces PfAMA1 specific antibody responses in rhesus macaques

To evaluate 293-gp96-Ig-PfCA vaccine-induced antibody responses in rhesus macaques, we tested pre- and post-immune sera (at effector and memory phase) by IFA and ELISA. Overall, most of the 293-gp96-Ig-PfCA vaccinated rhesus macaques did not have detectable levels of antibodies against sporozoites or PfCSP full-length protein (**Supplementary Figure S7A, C**). Only low levels of antibodies were detected against blood-stage parasites (Mean ± SD, Effector 516 ± 821, and Memory 113.75 ± 219.9) in the 293-gp96-Ig-PfCA group (**Supplementary Figure S7B**). The control DNA/Ad5-PfCA group elicited very high levels of antibodies against sporozoites, blood-stage parasites and PfCSP/PfAMA1 antigen during the effector phase, which decreased over time (**Supplementary Figures S7A–D**). Anti-PfAMA1 antibodies at both effector (P<0.01) and memory (P<0.01) phase were significantly higher in the DNA/Ad5-PfCA control group than the 293-gp96-Ig-PfCA vaccinated macaques (**Supplementary Figure S7D**). Notably, in 293-gp96-Ig-PfCA vaccinated macaques, anti-PfAMA1 antibody titers (effector, 4238.3 ± 3260.04, p <0.0001 and memory, 1767.25 ± 2148.87, p <0.001) were significantly higher compared to anti-PfCSP titers (effector, 49.8 ± 111.7 and memory, 44.25 ± 125.15) (**Supplementary Figures S7C, D**). 293-gp96-Ig mediated antibody responses are likely regulated by the type of immunogen, given the significant differences seen between PfCSP and PfAMA1 antibody responses in 293-gp96-Ig-PfCA vaccinated group.

## Discussion

*Plasmodium* liver-stage protection is believed to be associated with liver CD8+ tissue resident memory T (Trm) cells, which presumably act as a front-line defense against malaria infection (9). In the present study, we exploited unique properties of the cell based 293-gp96-Ig vaccines, to induce *P. falciparum-*specific memory CD8+ T cell responses in the liver of mice and rhesus macaques. Our results indicate that vaccination with HEK-293 cells transfected with gp96-Ig and the two malaria antigens; PfAMA1 and PfCSP (293-gp96-Ig-PfCA), can induce intrahepatic, effector CD8+ T cell responses and tissue resident memory CD8+ T cell responses. It is well established that the route of vaccination determines the quality and longevity of vaccine induced immune responses (49, 50). Induction of antigen-specific, 293-gp96-Ig mediated mucosal CD8+T cell responses depends on the type of antigen presenting/dendritic cells available at the site of vaccination (17, 19), which primes and activates T cells that home to epithelial tissues such as gut, reproductive tract and lungs (15, 17–19). Our data show that the subcutaneous route is the optimal and safest route for 293-gp96-Ig-PfCA administration to induce liver-specific CD8+ T cell responses against *Plasmodium* antigens (**Figures 2**, **3**, **S1**). Unlike previously described prime-and-trap or prime-and-target strategies (12, 14), 293-gp96-Ig-PfCA did not require any prior T cell priming as it induced a predominant population of liver-homing CD8+ T cells marked by Trm phenotype after a single dose (**Figure 2D**). Absence of circulating antigen-specific immune cells as early as one week post-last vaccination in the rhesus macaques agrees with the rapid liver-homing of the CD8+ T cells seen in mice (**Figures 2B**, **4**). *In situ* secretion of gp96-Ig could recruit and activate antigen-presenting cells to the local injection site, promote lymph node-independent cross-priming aided by co-stimulatory molecules and local expansion of CD8+ T cells (17, 42, 51). This clearly would bypass the secondary-lymphoid organ mediated T cell priming, allowing direct and rapid infiltration of T cells to the tissues targeted by the vaccination route. Preliminary data from previous studies suggests continued *in vivo* secretion of gp96 up to 5-7 days based on the survival of live, IP-injected allogeneic 3T3 fibroblasts secreting gp96 (16, 42, 51). This may result in an *in vivo* “depot effect” over several days, with continuous supply of antigenic-peptides, potentially chaperoned by gp96-Ig for cross-presentation and T cell priming. Therefore, the dual role performed by gp96-Ig, simultaneously activating antigen presenting cells (26) and chaperoning antigens for cross-presentation (52), are likely responsible for induction of the robust liver-homing CD8+ T cell responses we observed in our mouse studies. Our data suggest that the persistence of antigen-specific, liver memory CD8+ T cells induced by 293-gp96-Ig vaccination depends on the *Plasmodium* antigen and their quantity secreted by HEK-293 cells. Molecular interactions seem to be critical for selectivity and stability of the peptides bound by gp96 (53–56). Site-specific mutagenesis of the gp96 peptide binding pocket could alter and improve the gp96-peptide binding, indicating peptide selectivity regulated by molecular recognition (53–56). Thus, we believe that superior survival of PfCSP-specific memory responses over PfAMA1 seen in our mouse studies may be driven by the preferential binding of peptides by gp96-Ig and subsequent immunogenicity induce by the particular gp96-Ig-peptide complex. On the other hand, exhaustion phenotype on the liver Trm cells after whole parasite vaccination has been reported due to the extensive type I IFN signaling in CD8+ T cells (57). Even though we did not look at the PD-1 or LAG3 expression in our study, we previously reported controlled expression of type I IFNs by gp96-Ig that seems to be tightly regulated after infection (27).

The number of CD8+CD69+KLRG^low^ Trm cells have been shown to be associated with malaria liver-stage protection induced by prime-and-trap strategy (9). KLRG^low^ (“exKLRG1”) CD8+ T cells could differentiate into long-lived Trm cells, retain a high cytotoxic potential against influenza virus and a high proliferative capacity against tumor growth (43). Our gp96-Ig approach induced an exclusive population of CD8+CD69+KLRG1^low^ liver Trm cells in mice, thus indicating potential implications of gp96-Ig-vaccination in protection against malaria liver-stage infections. As reported in human liver CD8+ Trm cells, rhesus macaques also expressed CXCR3 phenotype in the majority of the hepatic CD8+CD69+ Trm cells immediately following both vaccinations (**Figure 5B**). CXCR3 plays a crucial role in initial liver recruitment of antigen-specific T cells (58, 59). CXCR3 ligands, CXCL9-11, upregulate in mouse liver sinusoidal endothelial cells (LSECs) and mediate effector CD8+ T cell homing to the liver during malaria liver-stage infection (58, 60, 61). In addition to liver-homing, CXCR3 signaling more likely is required for long-term retention of liver Trm cells. Supporting this hypothesis, we observed CXCR3 expression on resting CD8+CD69+ liver Trm cells up to 11 weeks in the vaccinated rhesus macaques, in the absence of vaccine antigens. The importance of CXCR3 signaling in maintenance of CD8+ Trm cells in genital mucosa has been previously shown in a mouse model of herpes simplex virus 2 (62). When re-challenged with cognate antigens, Trm cells appear to orchestrate a rapid effector response, in some cases by inducing CXCR3 ligands and by recruiting circulating T cells in an IFN-γ dependent manner (63). We believe that this may be true for the gp96-Ig approach as almost all the IFN-γ secreting liver CD8+CD69+ T cells co-expressed CXCR3+ upon antigen re-encounter. Also, the liver CD8+ Trm cells induced by the 293-gp96-Ig vaccination showed less cytolytic potential in the resting phase as well as after exposure to the cognate antigen *in vitro*, suggesting that cytotoxicity may not play a major role in recall memory responses after 293-gp96-Ig vaccination. The reduced cytotoxic function of liver CD8+CD69+ Trm cells compared to circulating and intrahepatic effector T cells have been previously documented (11, 64–67). Liver Trm may potentially down-regulate the capacity to induce immediate cytotoxicity, limiting T cell-mediated pathology in healthy liver (10). Granzyme-B+ Trm were found to be enriched during CXCR3+ chronic hepatitis B infection, antagonizing tolerance and adding to liver damage (66, 67). Whether the memory phase responses characterized by an increased fraction of granzyme B+ Trm cells in DNA/Ad5-PfCA vaccination would lead to tolerance is not known.

While CD103, a receptor that localizes cells to epithelial connections (9, 46), did not express substantially in both mice and rhesus macaques after 293-gp96-Ig-PfCA vaccination, DNA/Ad5-PfCA vaccination induced CD103 expression (**Figure 5F**). In human liver, a subset of Trm cells that co-expresses CD103 was found to be enriched in patients with chronic hepatitis B, possibly causing portal infiltration and liver pathology (68). Even though we found a subset of CXCR3+ Trm cells in DNA/Ad5-PfCA vaccinated animals, we did not observe any liver pathology.

We have found IL-2 producing PfCA-specific CD8+ T cells in the liver during the 293-gp96-Ig-PfCA vaccination memory phase (**Figure 6B****)**. As reported in the Pellet study (10), this unusually high IL-2 production is likely critical to the protective potential of hepatic CD8+ Trm cells. They showed that liver-resident hepatitis B virus-specific CD8+ T cells containing high levels of IL-2 are associated with viral control. CD8+ T cells need to make their own IL-2 in order to drive persistent memory responses with adequate IFN-γ production (69, 70) and this could be particularly relevant for the maintenance of memory in tissues like liver, where CD4+ T cell frequency is low (69). Importantly they also confirmed that by sequential exposure of PBMCs to IL-15 and TGFβ and/or T cell Receptor (TCR) engagement one could induce liver-resident CD8+ T cells.

Obvious limitation of nonhuman primate portion of this study is the low number of analyzed samples (two macaques) and the lack of statistical tests that can identify significant relationships or connections within a given data set. However, analyzed data of two nonhuman primates confirms the mouse findings and supports the main idea of this study that secreted heat shock protein based gp96-Ig-PfCA malaria vaccine induces antigen specific intrahepatic CD8+ T cell responses in different species. Given the lack of robust *ex vivo* and *in vitro* assays to functionally examine human immune responses to Plasmodium infection and immunization with candidate vaccines, our study, originally designed as exploratory study for intrahepatic analysis in nonhuman primates, will provide valuable information to all future studies that are exploring vaccine induced intrahepatic CD8+ T cell responses.

In summary, this is the first report describing the secreted heat shock protein cell-based vaccine approach that induces malaria-specific Trm CD8+ T cell responses positioned to maintain hepatic immunosurveillance and exert rapid front-line pathogen defense.

## Data availability statement

The raw data supporting the conclusions of this article will be made available by the authors, without undue reservation.

## Ethics statement

Non-human primate research work was conducted under an approved animal use protocol in an AAALAC International-accredited facility at the Walter Reed Army Institute of Research, Silver Spring, MD in compliance with the Animal Welfare Act and all other federal statutes and regulations relating to animals and experiments involving animals, and adheres to principles stated in the Guide for Care and Use of Laboratory Animals, NRC Publication, 2011 edition.

## Author contributions

NS and EV obtained funding, conceived and coordinated experiments. KE coordinated the funds and experiments, and assisted with manuscript preparations. LP, NS, WW, NP conducted the experiments, analyzed and interpreted the data and wrote the manuscript. EF, KR, DG provided expert support on samples processing, flow cytometry data acquisition/analysis/interpretation, immunofluorescence tissue staining and reviewed the manuscript. MS, HG and JH process NHP study biosamples and conducted the FluoroSpot Assays and related data analyses. TR provided support on PfCSP/PfAMA1 ELISA planning and data acquisition. FB and MM were the subject matter experts for animal care and use and assisted in all non-human primate procedures including anesthesia and organ harvests. MH provided surgical and pathology expertise as the key personnel excising all non-human primate organs. All authors contributed to the article and approved the submitted version.

## Author disclaimer

The views expressed in this article reflect the results of research conducted by the author and do not necessarily reflect the official policy or position of the Henry M. Jackson Foundation for the Advancement of Military Medicine, Inc., Department of Army, Department of the Navy, Department of Defense, nor the U.S. Government. KAE, FAB, MAH, and MLM are military service members and MS and EV are employees of the U.S. Government. This work was prepared as part of their official duties. Title 17, U.S.C., §105 provides that ‘copyright protection under this title is not available for any work of the U.S. Government.’ Title 17 U.S.C. §101 defines a U.S. Government work as a work prepared by a military service member or employee of the U.S. Government as part of that person’s official duties. Any opinions, findings and conclusions or recommendations expressed in this material are those of the author(s) and do not necessarily reflect the views of the Naval Medical Research Center and the Naval Medical Logistics Command. Material has been reviewed by the Walter Reed Army Institute of Research. There is no objection to its presentation and/or publication. The opinions or assertions contained herein are the private views of the author, and are not to be construed as official, or as reflecting true views of the Department of the Army or the Department of Defense. Research was conducted under an approved animal use protocol in an AAALAC International-accredited facility in compliance with the Animal Welfare Act and all other federal statutes and regulations relating to animals and experiments involving animals, and adheres to principles stated in the Guide for Care and Use of Laboratory Animals, NRC Publication, 2011 edition.
