# Supplementary material for: Induction of antigen specific intrahepatic CD8+ T cell responses by a secreted heat shock protein based gp96-Ig-PfCA malaria vaccine
Source: Front Immunol. 2023 Mar 28;14:1130054. doi: 10.3389/fimmu.2023.1130054 (PMC10086177; doi:10.3389/fimmu.2023.1130054)
Supplement: Supplementary file 1 [file Presentation_1.pptx]

## Slide 1
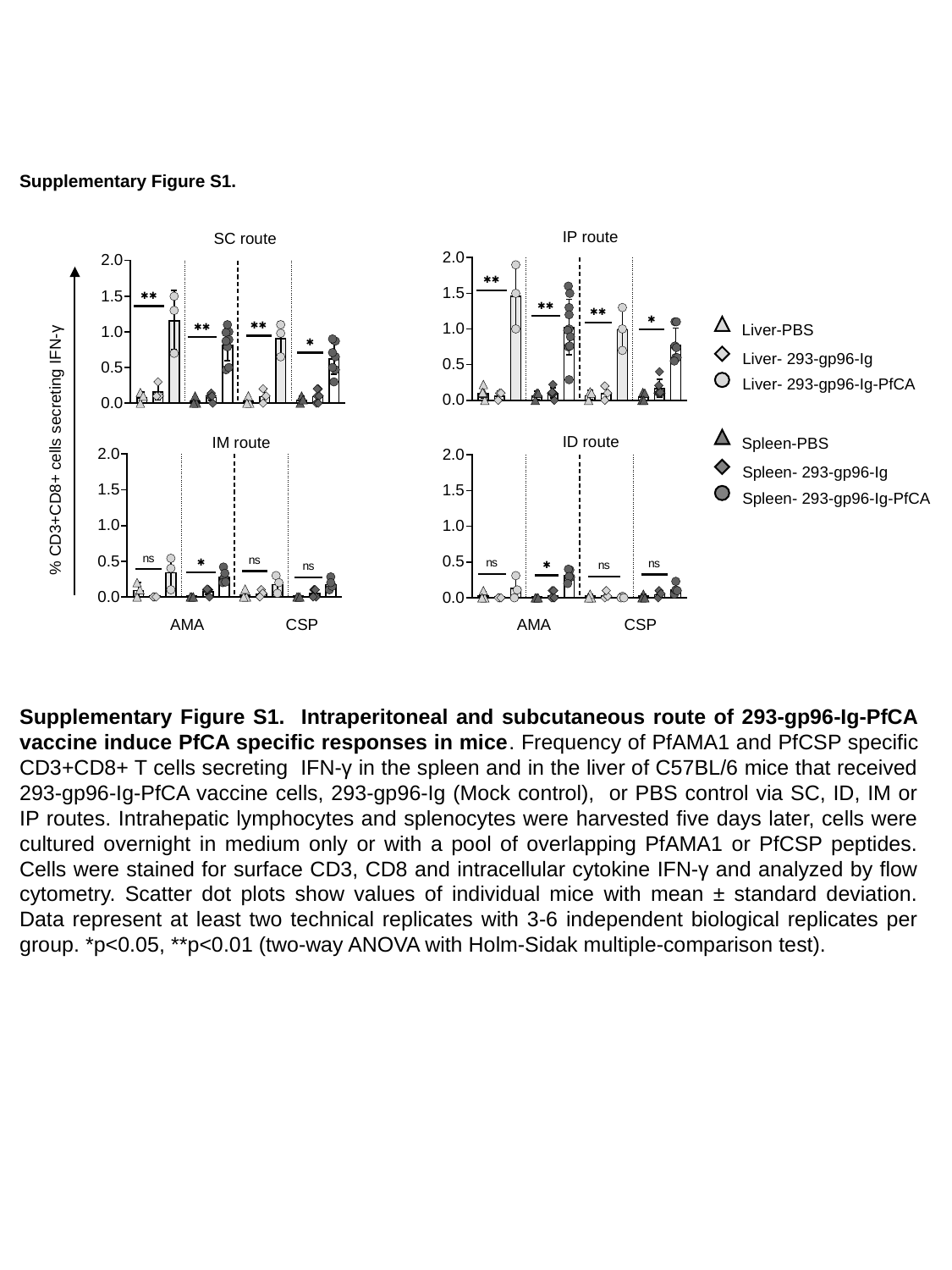

Supplementary Figure S1.
IP route
SC route
% CD3+CD8+ cells secreting IFN-γ
ID route
IM route
AMA
CSP
AMA
CSP
Liver-PBS
Liver- 293-gp96-Ig
Liver- 293-gp96-Ig-PfCA
Spleen-PBS
Spleen- 293-gp96-Ig
Spleen- 293-gp96-Ig-PfCA
Supplementary Figure S1. Intraperitoneal and subcutaneous route of 293-gp96-Ig-PfCA vaccine induce PfCA specific responses in mice. Frequency of PfAMA1 and PfCSP specific CD3+CD8+ T cells secreting IFN-γ in the spleen and in the liver of C57BL/6 mice that received 293-gp96-Ig-PfCA vaccine cells, 293-gp96-Ig (Mock control), or PBS control via SC, ID, IM or IP routes. Intrahepatic lymphocytes and splenocytes were harvested five days later, cells were cultured overnight in medium only or with a pool of overlapping PfAMA1 or PfCSP peptides. Cells were stained for surface CD3, CD8 and intracellular cytokine IFN-γ and analyzed by flow cytometry. Scatter dot plots show values of individual mice with mean ± standard deviation. Data represent at least two technical replicates with 3-6 independent biological replicates per group. *p<0.05, **p<0.01 (two-way ANOVA with Holm-Sidak multiple-comparison test).

## Slide 2
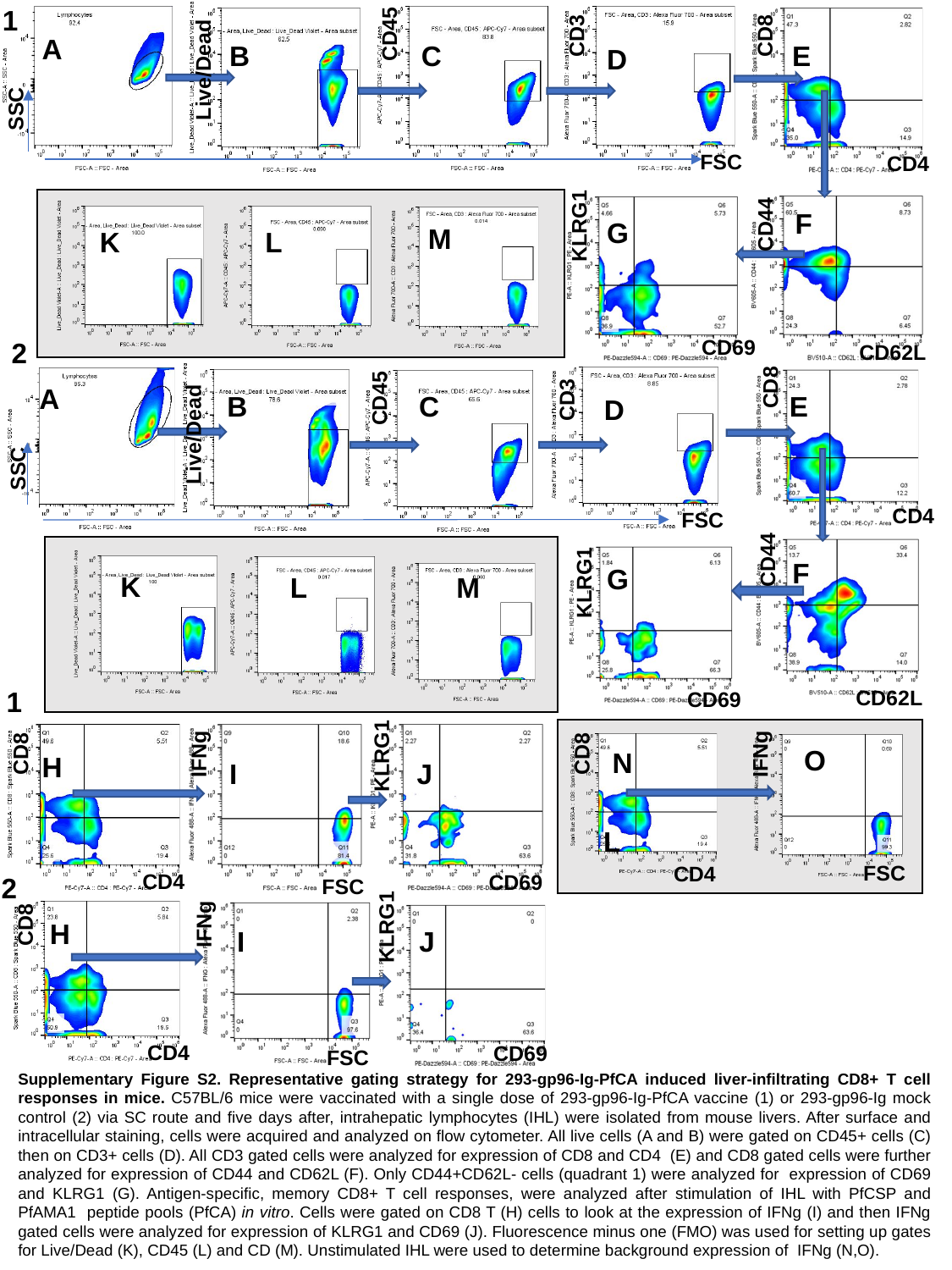

1
CD3
CD8
CD45
A
B
E
C
D
Live/Dead
SSC
FSC
CD4
CD44
F
KLRG1
G
M
K
L
CD69
2
CD62L
CD8
CD3
CD45
A
E
C
B
D
Live/Dead
SSC
CD4
FSC
CD44
F
G
KLRG1
K
M
L
CD62L
1
CD69
CD8
CD8
IFNg
IFNg
KLRG1
O
N
H
I
J
L
FSC
CD4
CD4
CD69
2
FSC
CD8
IFNg
KLRG1
H
J
I
CD4
CD69
FSC
Supplementary Figure S2. Representative gating strategy for 293-gp96-Ig-PfCA induced liver-infiltrating CD8+ T cell responses in mice. C57BL/6 mice were vaccinated with a single dose of 293-gp96-Ig-PfCA vaccine (1) or 293-gp96-Ig mock control (2) via SC route and five days after, intrahepatic lymphocytes (IHL) were isolated from mouse livers. After surface and intracellular staining, cells were acquired and analyzed on flow cytometer. All live cells (A and B) were gated on CD45+ cells (C) then on CD3+ cells (D). All CD3 gated cells were analyzed for expression of CD8 and CD4 (E) and CD8 gated cells were further analyzed for expression of CD44 and CD62L (F). Only CD44+CD62L- cells (quadrant 1) were analyzed for expression of CD69 and KLRG1 (G). Antigen-specific, memory CD8+ T cell responses, were analyzed after stimulation of IHL with PfCSP and PfAMA1 peptide pools (PfCA) in vitro. Cells were gated on CD8 T (H) cells to look at the expression of IFNg (I) and then IFNg gated cells were analyzed for expression of KLRG1 and CD69 (J). Fluorescence minus one (FMO) was used for setting up gates for Live/Dead (K), CD45 (L) and CD (M). Unstimulated IHL were used to determine background expression of IFNg (N,O).

## Slide 3
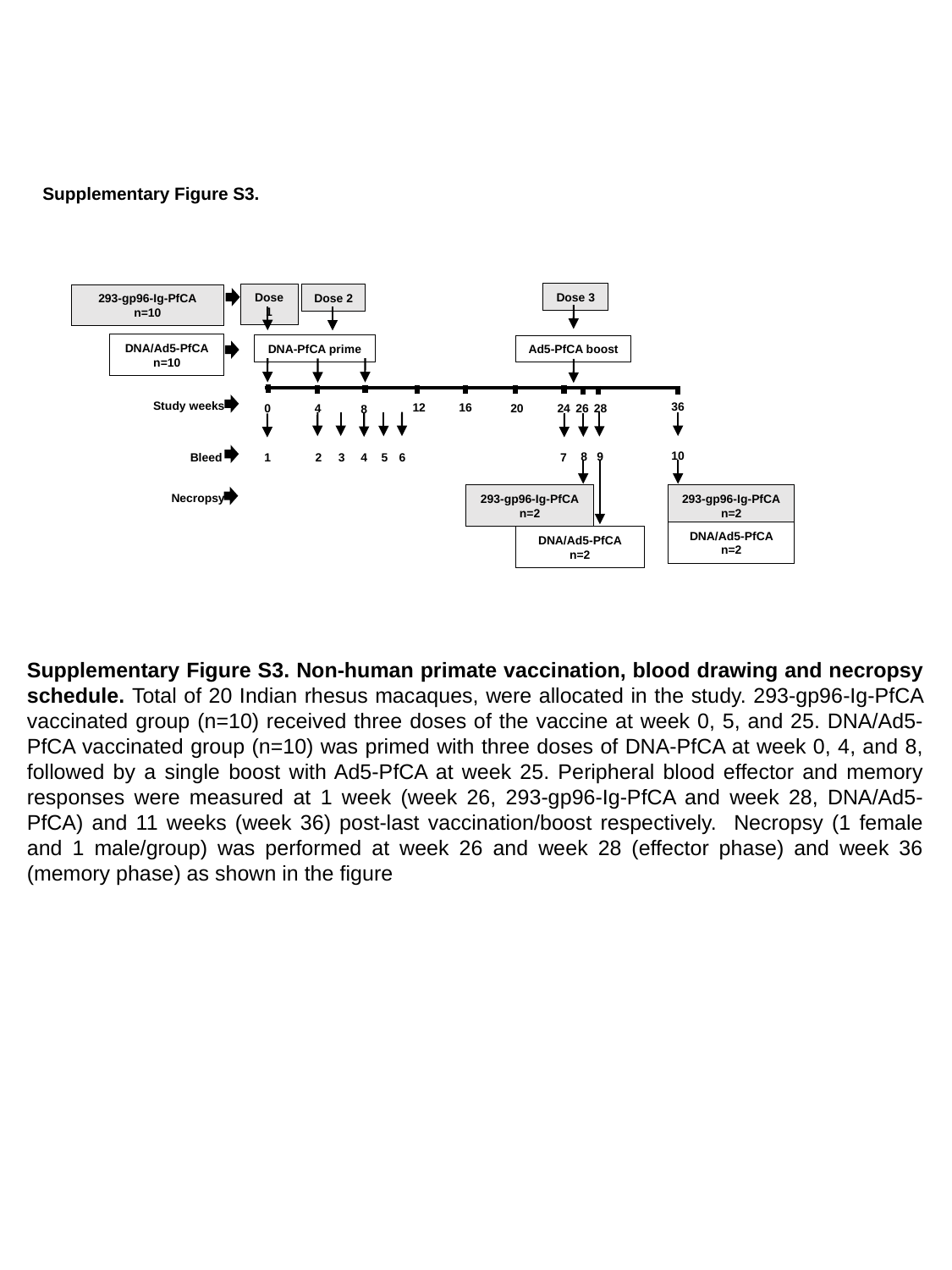

Supplementary Figure S3.
Dose 3
Dose 1
Dose 2
293-gp96-Ig-PfCA
n=10
DNA/Ad5-PfCA
n=10
DNA-PfCA prime
Ad5-PfCA boost
Study weeks
36
16
12
0
28
4
20
24
26
8
2
3
4
5
6
1
7
10
9
8
Bleed
Necropsy
293-gp96-Ig-PfCA
n=2
293-gp96-Ig-PfCA
n=2
DNA/Ad5-PfCA
n=2
DNA/Ad5-PfCA
n=2
Supplementary Figure S3. Non-human primate vaccination, blood drawing and necropsy schedule. Total of 20 Indian rhesus macaques, were allocated in the study. 293-gp96-Ig-PfCA vaccinated group (n=10) received three doses of the vaccine at week 0, 5, and 25. DNA/Ad5-PfCA vaccinated group (n=10) was primed with three doses of DNA-PfCA at week 0, 4, and 8, followed by a single boost with Ad5-PfCA at week 25. Peripheral blood effector and memory responses were measured at 1 week (week 26, 293-gp96-Ig-PfCA and week 28, DNA/Ad5-PfCA) and 11 weeks (week 36) post-last vaccination/boost respectively. Necropsy (1 female and 1 male/group) was performed at week 26 and week 28 (effector phase) and week 36 (memory phase) as shown in the figure

## Slide 4
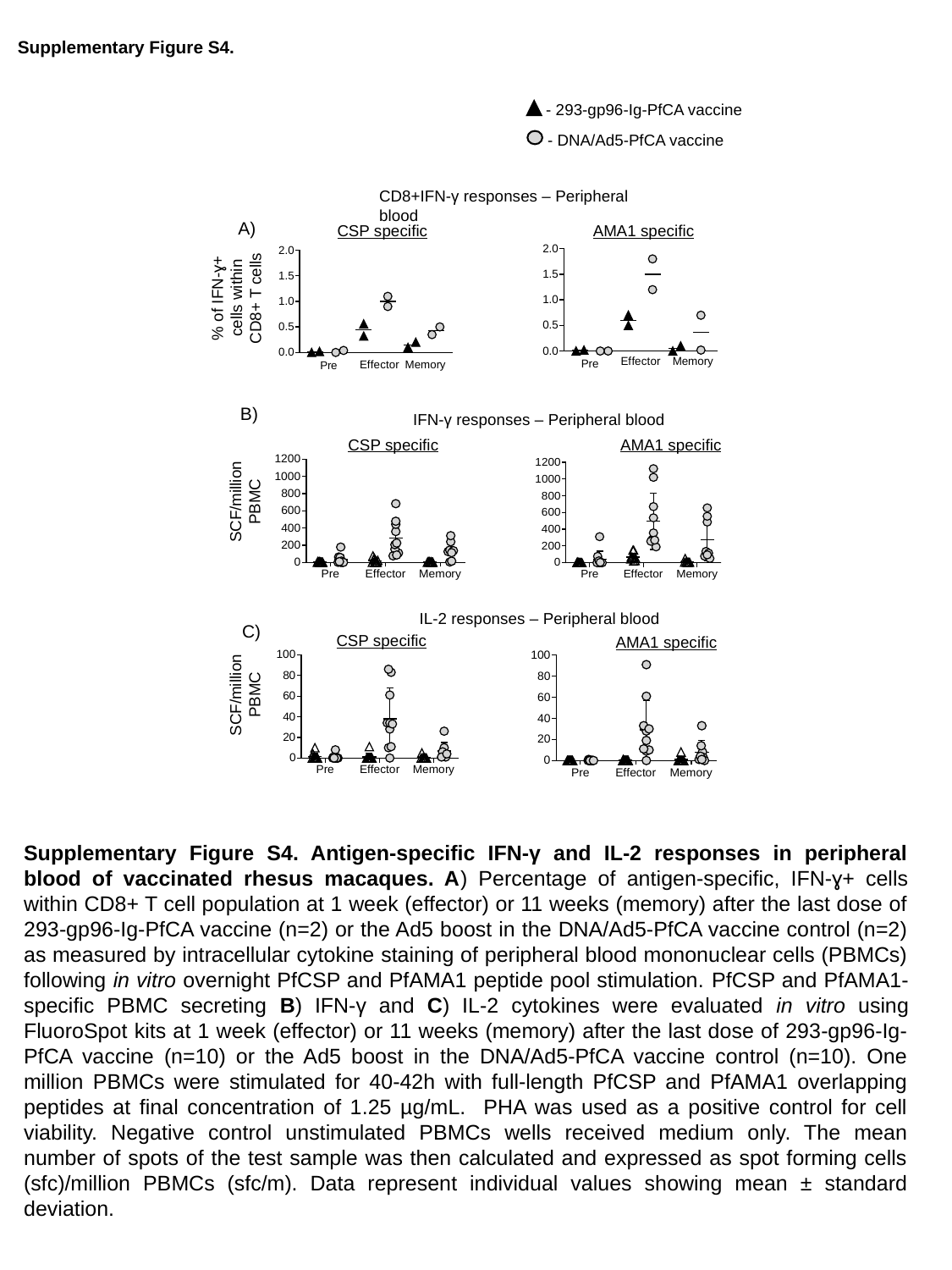

Supplementary Figure S4.
- 293-gp96-Ig-PfCA vaccine
 - DNA/Ad5-PfCA vaccine
CD8+IFN-γ responses – Peripheral blood
A)
CSP specific
AMA1 specific
% of IFN-ɣ+ cells within CD8+ T cells
B)
IFN-γ responses – Peripheral blood
AMA1 specific
CSP specific
SCF/million PBMC
IL-2 responses – Peripheral blood
C)
CSP specific
SCF/million PBMC
AMA1 specific
Supplementary Figure S4. Antigen-specific IFN-γ and IL-2 responses in peripheral blood of vaccinated rhesus macaques. A) Percentage of antigen-specific, IFN-ɣ+ cells within CD8+ T cell population at 1 week (effector) or 11 weeks (memory) after the last dose of 293-gp96-Ig-PfCA vaccine (n=2) or the Ad5 boost in the DNA/Ad5-PfCA vaccine control (n=2) as measured by intracellular cytokine staining of peripheral blood mononuclear cells (PBMCs) following in vitro overnight PfCSP and PfAMA1 peptide pool stimulation. PfCSP and PfAMA1-specific PBMC secreting B) IFN-γ and C) IL-2 cytokines were evaluated in vitro using FluoroSpot kits at 1 week (effector) or 11 weeks (memory) after the last dose of 293-gp96-Ig-PfCA vaccine (n=10) or the Ad5 boost in the DNA/Ad5-PfCA vaccine control (n=10). One million PBMCs were stimulated for 40-42h with full-length PfCSP and PfAMA1 overlapping peptides at final concentration of 1.25 µg/mL. PHA was used as a positive control for cell viability. Negative control unstimulated PBMCs wells received medium only. The mean number of spots of the test sample was then calculated and expressed as spot forming cells (sfc)/million PBMCs (sfc/m). Data represent individual values showing mean ± standard deviation.

## Slide 5
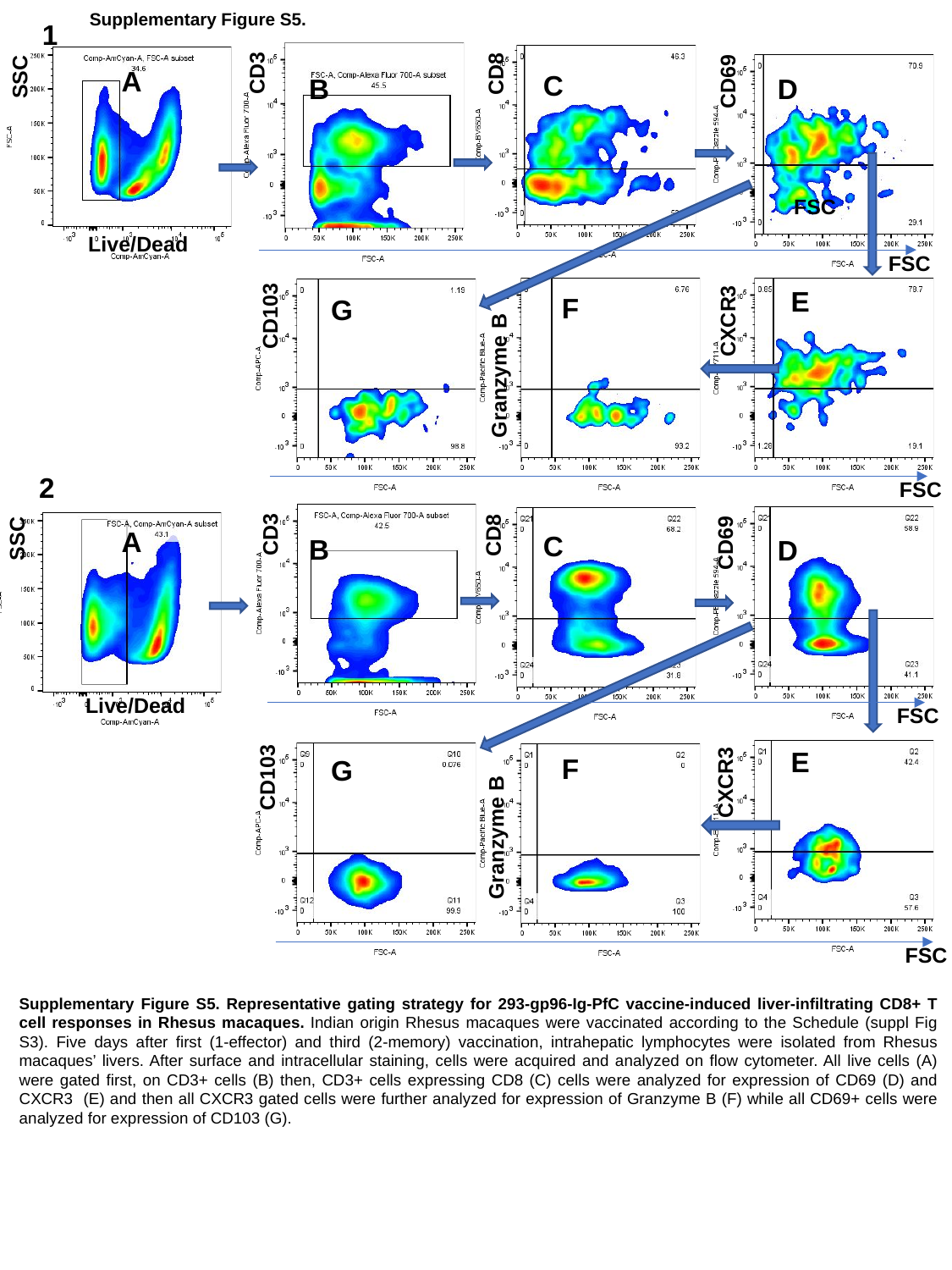

Supplementary Figure S5.
1
CD3
SSC
CD8
CD69
A
C
B
D
FSC
Live/Dead
FSC
CD103
E
F
G
CXCR3
Granzyme B
2
FSC
CD3
SSC
CD8
CD69
A
C
B
D
Live/Dead
FSC
CD103
E
F
G
CXCR3
Granzyme B
FSC
Supplementary Figure S5. Representative gating strategy for 293-gp96-Ig-PfC vaccine-induced liver-infiltrating CD8+ T cell responses in Rhesus macaques. Indian origin Rhesus macaques were vaccinated according to the Schedule (suppl Fig S3). Five days after first (1-effector) and third (2-memory) vaccination, intrahepatic lymphocytes were isolated from Rhesus macaques’ livers. After surface and intracellular staining, cells were acquired and analyzed on flow cytometer. All live cells (A) were gated first, on CD3+ cells (B) then, CD3+ cells expressing CD8 (C) cells were analyzed for expression of CD69 (D) and CXCR3 (E) and then all CXCR3 gated cells were further analyzed for expression of Granzyme B (F) while all CD69+ cells were analyzed for expression of CD103 (G).

## Slide 6
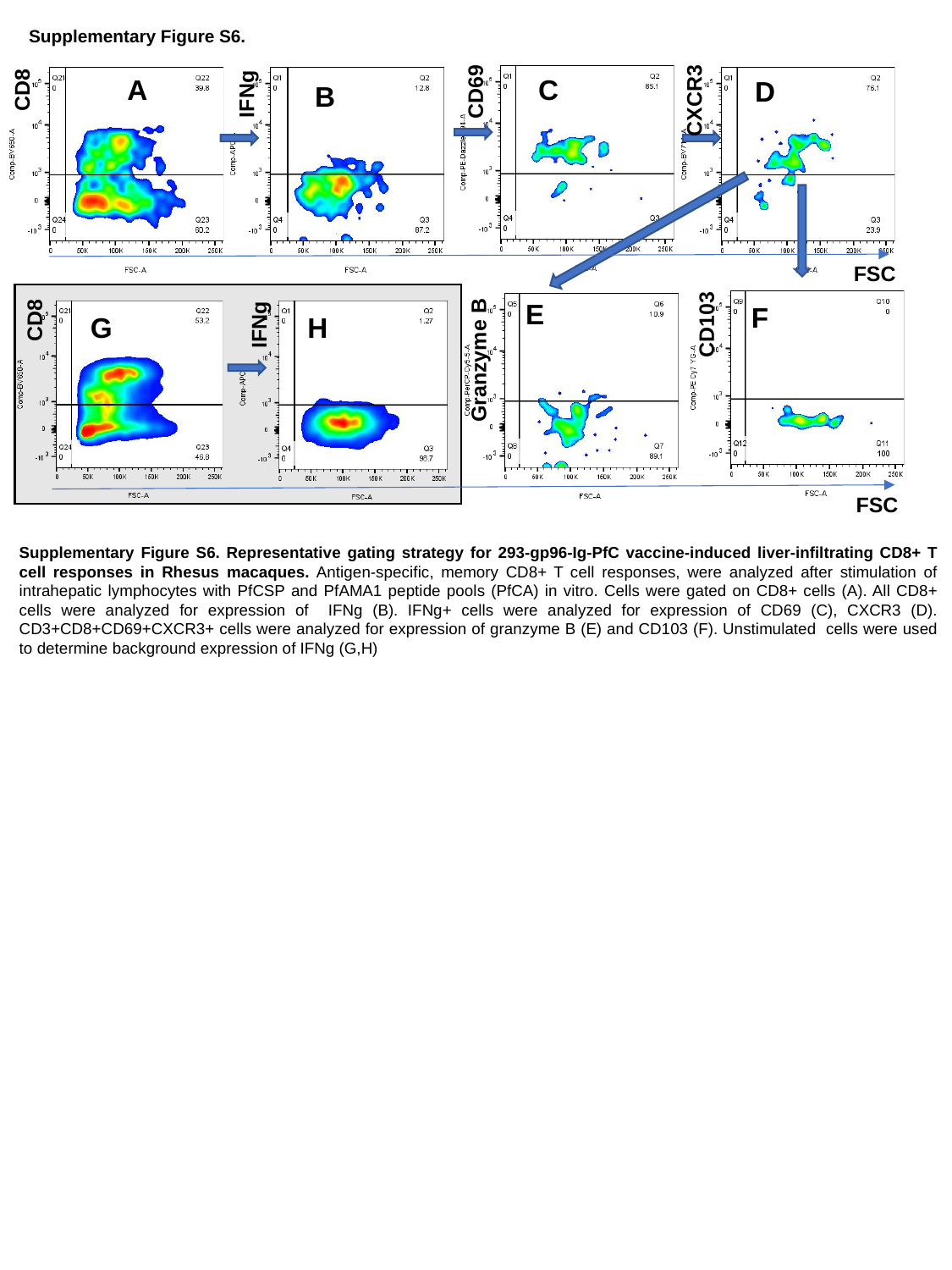

Supplementary Figure S6.
CD8
CD69
IFNg
A
C
D
B
CXCR3
FSC
CD103
CD8
E
IFNg
F
H
G
Granzyme B
FSC
Supplementary Figure S6. Representative gating strategy for 293-gp96-Ig-PfC vaccine-induced liver-infiltrating CD8+ T cell responses in Rhesus macaques. Antigen-specific, memory CD8+ T cell responses, were analyzed after stimulation of intrahepatic lymphocytes with PfCSP and PfAMA1 peptide pools (PfCA) in vitro. Cells were gated on CD8+ cells (A). All CD8+ cells were analyzed for expression of IFNg (B). IFNg+ cells were analyzed for expression of CD69 (C), CXCR3 (D). CD3+CD8+CD69+CXCR3+ cells were analyzed for expression of granzyme B (E) and CD103 (F). Unstimulated cells were used to determine background expression of IFNg (G,H)

## Slide 7
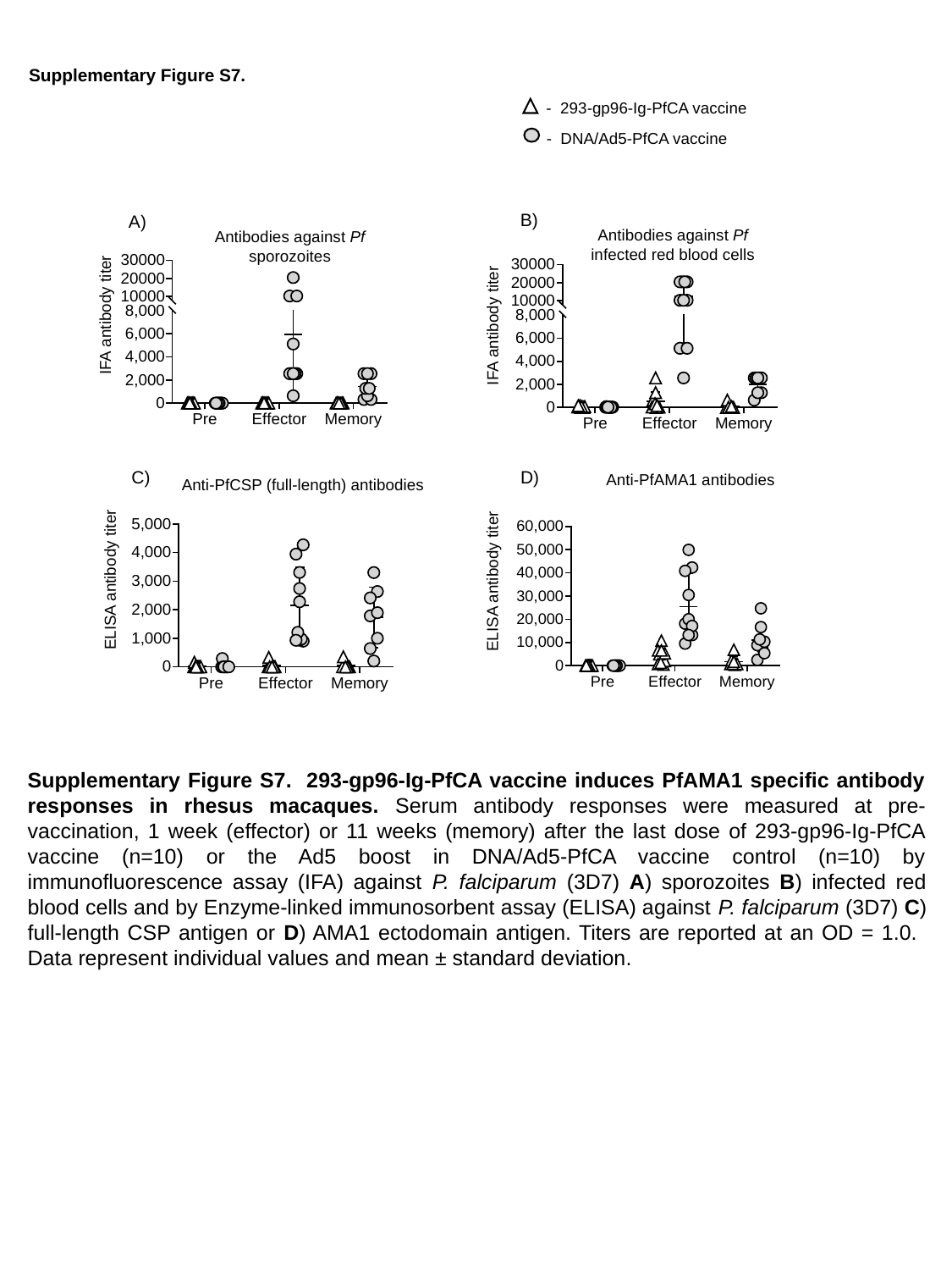

Supplementary Figure S7.
 - 293-gp96-Ig-PfCA vaccine
 - DNA/Ad5-PfCA vaccine
Antibodies against Pf infected red blood cells
IFA antibody titer
B)
A)
Antibodies against Pf sporozoites
IFA antibody titer
C)
D)
Anti-PfAMA1 antibodies
ELISA antibody titer
Anti-PfCSP (full-length) antibodies
ELISA antibody titer
Supplementary Figure S7. 293-gp96-Ig-PfCA vaccine induces PfAMA1 specific antibody responses in rhesus macaques. Serum antibody responses were measured at pre-vaccination, 1 week (effector) or 11 weeks (memory) after the last dose of 293-gp96-Ig-PfCA vaccine (n=10) or the Ad5 boost in DNA/Ad5-PfCA vaccine control (n=10) by immunofluorescence assay (IFA) against P. falciparum (3D7) A) sporozoites B) infected red blood cells and by Enzyme-linked immunosorbent assay (ELISA) against P. falciparum (3D7) C) full-length CSP antigen or D) AMA1 ectodomain antigen. Titers are reported at an OD = 1.0. Data represent individual values and mean ± standard deviation.
